# From End to End: Gaining, Sorting, and Employing High-Density Neural Single Unit Recordings

**DOI:** 10.3389/fninf.2022.851024

**Published:** 2022-06-13

**Authors:** Réka Barbara Bod, János Rokai, Domokos Meszéna, Richárd Fiáth, István Ulbert, Gergely Márton

**Affiliations:** ^1^Laboratory of Experimental Neurophysiology, Department of Physiology, Faculty of Medicine, George Emil Palade University of Medicine, Pharmacy, Science and Technology of Târgu Mureş, Târgu Mureş, Romania; ^2^Integrative Neuroscience Group, Institute of Cognitive Neuroscience and Psychology, Research Centre for Natural Sciences, Budapest, Hungary; ^3^School of PhD Studies, Semmelweis University, Budapest, Hungary; ^4^Faculty of Information Technology and Bionics, Pázmány Péter Catholic University, Budapest, Hungary

**Keywords:** spike sorting, single unit recordings, neural sensors, clustering, algorithm evaluation

## Abstract

The meaning behind neural single unit activity has constantly been a challenge, so it will persist in the foreseeable future. As one of the most sourced strategies, detecting neural activity in high-resolution neural sensor recordings and then attributing them to their corresponding source neurons correctly, namely the process of spike sorting, has been prevailing so far. Support from ever-improving recording techniques and sophisticated algorithms for extracting worthwhile information and abundance in clustering procedures turned spike sorting into an indispensable tool in electrophysiological analysis. This review attempts to illustrate that in all stages of spike sorting algorithms, the past 5 years innovations' brought about concepts, results, and questions worth sharing with even the non-expert user community. By thoroughly inspecting latest innovations in the field of neural sensors, recording procedures, and various spike sorting strategies, a skeletonization of relevant knowledge lays here, with an initiative to get one step closer to the original objective: deciphering and building in the sense of neural transcript.

## Introduction

Electrophysiology has been constructed on electrical properties of biological membranes provided by ion exchanges between extra- and intracellular fluids. This phenomenon gives rise to the presence of electrochemical gradient across every single eukaryotic cell membrane, the state of net equilibrium called resting membrane potential. Transitory perturbations of this balance-like state and the spillover feature of these sudden changes render excitable cells, such as neurons capable of electrochemical signal propagation. An action potential (AP) occurs when the resting membrane potential of a neuron, around −70 mV, is reversed toward positive values in about an ms and then restored (Raghavan et al., 2019). Regarded as a foundation stone in neurophysiology, recorded extracellular APs (commonly referred to as spikes) are the fingerprints of single neurons' activities, an observation that has been fueling neuroscientific research for almost a century (Carlson and Carin, 2019; Zhang and Constandinou, 2021a). Analyzing spike trains and spatiotemporal properties of extracellular AP waveforms provides us precious evidence of a cell's functional profile and morphology, including dendritic tree architecture, surrounding environment, and relative position of the recording site (Chaure et al., 2018; Rodriguez-Collado and Rueda, 2021; Soleymankhani and Shalchyan, 2021) and sheds light on the meticulously orchestrated functioning of neural networks (Leibig et al., 2016; Luan et al., 2018). Besides providing insight into brain activity at the highest temporal resolution currently available (Rey et al., 2015; Wouters et al., 2021), facilitating the “reverse-engineering” of the brain (Petrantonakis and Poirazi, 2017), extracellular APs are eagerly sourced in the development of brain-machine interfaces, too (Hammad et al., 2016).

**Figure 1:**
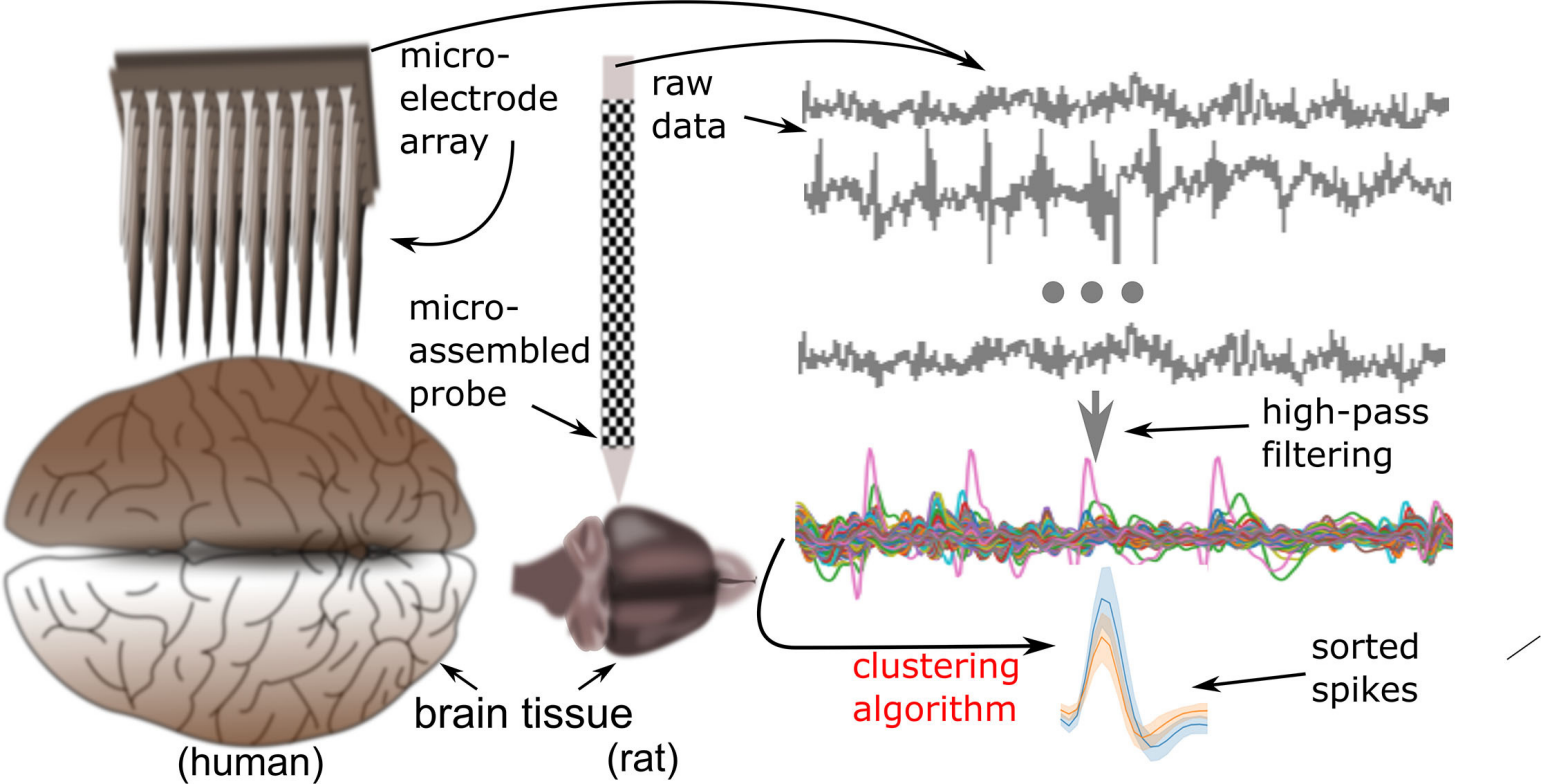
From brain to spike. Gaining, sorting, and employing of spikes begin with the acquisition of neural signals, either from human or animal neural tissues. Microelectrode arrays and micro-assembled probes are two of the most frequently used modalities of neural tissue recordings. These recordings are routinely filtered in order to render them more accessible for the conventional spike sorting procedure. As a result, single unit activities would be recognized and finally organized into clusters based on similar morphology.

However, barely collecting APs does not reveal much information on representation among neural populations, activity correlations, let alone higher order brain functions (Lefebvre et al., 2016; Abbott et al., 2020; Valencia and Alimohammad, 2021). To bridge the gap between a signal [i.e., voltage change recorded as waveform or, ubiquitously speaking, a spike (Dallal et al., 2016)] and its actual meaning, one must detect neural activity and attribute it to its corresponding source neuron correctly, a process named spike sorting (Pachitariu et al., 2016; Pakman et al., 2020; Guzman et al., 2021; Pagin, 2021) (Figure 1). As it is commonly treated, at the heart of the spike sorting technique lies a clustering problem (Souza et al., 2019), but prior steps such as the subsequently presented spike detection, feature extraction, and alignment are inescapable aspects as well (Steinmetz, 2017).

It is enough to look back on half a decade's spike sorting techniques to demonstrate the urging need for more efficient algorithms (Rossant et al., 2016). With the ever-growing number of recording sites capable of detecting thousands of APs simultaneously and computational power in our hands (Navratilova et al., 2016; Haessig et al., 2020), we must turn our attention to challenges that lay still unresolved or require fine-tuning (Mena et al., 2017; Zhang et al., 2018; Jurczynski et al., 2021).

In this study, we aimed to outline up-to-date improvements in the field of extracellular neurophysiological data recordings and then present and compare the most elaborate and promising approaches in each spike sorting step. Next, we described some pivotal points of spike sorting that we consider worthy of enhancement. Finally, a set of probable further directions was summarized.

## Data Acquisition: From Single Electrodes to Neuropixels Probes

Being aware of Moore's adapted law on neural recording devices, we might expect that the number of channels in which we can record simultaneously practically doubles every 7 years (Radmanesh et al., 2021). In light of this, we are inclined to expect that the number of spikes recorded should likewise grow steeply. For this assumption to be true, a series of circumstances should also be realized, such as our recording devices provide a decent signal-to-noise ratio and provide a quality that ultimately enables rejecting artifacts in a stable manner.

Neural sensors should be able to detect voltage variations on a large scale (Saggese et al., 2021). Each electrode records by default extracellular de- and hyperpolarizations in the close vicinity of its nano-to-micrometer-wide tip (to a distance of about 140 μm in the case of a single wire electrode), and given the propagating nature of action potentials, these deflections would not only be APs assigned to a particular neuron, or single unit activity (SUA) but rather the spatio-temporal summation of a neural population in the close proximity, multi-unit activities (MUAs), and local field potentials (Hong and Lieber, 2019; Tambaro et al., 2021). Most spike sorting techniques discard local field potentials by simply high pass filtering data and concentrate on the spatial and temporal contexts of signal propagation (Abbott et al., 2020), although invasive brain-machine interface (BMI) systems could also possibly profit from this frequency range (Hammad et al., 2016).

### Biocompatibility and Physical Considerations

To increase the quality of neurophysiological recordings, it seems to be a good idea to improve the biocompatibility of the recording tool, which means that its physical and chemical properties should approximate the ones of the intact neural tissue. One step forward could be an electrode coating or insulation with a boron-doped diamond material (Klempír et al., 2020) or polyethylene glycol (PEG) 4000 for increasing the rigidity of ultra-flexible probes (Guan et al., 2019; Vasileva and Bondar, 2021). Another promising material that unites strength and flexibility on a neural probe scale is carbon fiber, with increasing attentiveness in neurophysiological studies (Cetinkaya et al., 2018). Another procedure that decreases damage during implantation is the slow insertion of the recording devices. For even better results, administration of certain steroidal anti-inflammatory drugs or vasoconstrictive pharmakons, dura mater preparation and blood vessel-sparing penetration may be attempted (Fiáth et al., 2019a; He et al., 2020). Shank volume reduction is also proportional with tissue damage reduction (Musk, 2019). Nevertheless, elasticity has its own drawbacks when surgical procedures are considered; therefore, stiffening supports or even implantation shuttles can be of benefit (Wang et al., 2021). The latter solution would be of key importance when implantation targets dictate a high precision or vascular structures should be exceptionally spared (Fiani et al., 2021).

There has been serious aspirations to decrease electrode impedance by applying special coatings to recording probes, for instance gold plating and polyethylene glycol additives (Kuperstein, 2021), poly (3,4-ethylenedioxythiophene) (PEDOT) (Saunier et al., 2020), or polystyrene sulfonate (Neto et al., 2018). However, it turned out that impedance control is of less importance when conceptualizing an electrode (Neto et al., 2018). Furthermore, impedance can be diminished by simply growing the surface of contacts, possibly entailing signal dissipation and decrease in amplitude (Camuñas-Mesa and Quiroga, 2013). Another more relevant aspect is that when recording potentials of interest, one should be concerned about the reference either theoretically set at zero potential or positioned far enough to be considered at least uncorrelated from recording probes (Jurczynski et al., 2021). Electrode geometry or channel density can also raise non-trivial surgical questions (Rivnay et al., 2017) but more importantly influence the quality of recordings, namely, recording sites located closer to the border of rigid silicon shanks are prone to s with a higher signal-to-noise ratio (Fiáth et al., 2021). Implementation of wider shanks has its two-faced features, too: the more volume covered and neural activity observed by the electrode, the more significant tissue scarring will be (Tóth et al., 2021).

### Neural Sensors

Progress in electrode fabrication has not only yielded to multiply the amount of recording sites at a fast pace (Kim et al., 2018) but also finds the most suitable means for any electrophysiological experiment. Take for example 3D self-rolled biosensor arrays that are meant to collect data from three-dimensional cortical spheroid cultures (Kalmykov et al., 2021), with their high-density channel profile, Neuropixels probes are ideal for functional connectivity studies (Wang et al., 2021). In the following paragraphs, some of the most common sensor types used for electrophysiological recordings are presented.

#### Single Electrodes

Neural recordings may flawlessly be acquired by single, glass, or coated microwires, but their usefulness hinge on the tested experimental hypothesis. Whether dense-packed structures, for instance pyramidal cells of the hippocampus, are under investigation, plus population activity as local field potential brings sufficient information, individual electrodes constitute an ideal choice (Rutishauser et al., 2006). On the other hand, it is also evident that single unit activity can hardly be relieved by a single electrode recording because of the abundance of independently firing nearby neurons, without any knowledge of their spatial coordinates (Petrantonakis and Poirazi, 2017).

#### Tetrodes

The ensemble of four closely packed electrodes with the purpose of recording extracellular potentials defines a tetrode. Tetrodes may originate from simple copper wires (Lu et al., 2018) to even the more refined gold covering (Kuperstein, 2021) or quartz-coated platinum-tungsten alloy (Ravikumar, 2021), and their superiority compared to single electrodes stands in allowing to differentiate waveforms belonging to distinct neurons, thanks to their spatial configuration (Rey et al., 2015). Even so, tetrodes fall short when extracellular AP distributions must be investigated considering a complex set of attributes, such as distributions of multidimensional extracellular potentials. One should also bear in mind that tetrode usage as a recording facility technically culminates in animal studies (Vasileva and Bondar, 2021), since human applications are sparse (Despouy et al., 2020).

#### Polytrodes

Grading up from tetrodes, polytrodes, typically insulated metal wires (Francoeur et al., 2021) or micro-assembled silicon probes, have increased exponentially the number of neurons in a single observation (Neto et al., 2016). With a channel count ranging from 8 to 64, each one set 25–200 μm apart and arranged in 1–3 columns, U-/V- or S-probes are reliable tools for study on different cortical layers (Wang et al., 2021). As the time span of recorded data is increasingly substantial, it is recommended that beginning with polytrodes, data should be split into subsets, in a divide-and-conquer fashion (Swindale and Spacek, 2014; Lee et al., 2017; Diggelmann et al., 2018). Besides the probes by Plexon described above, there have been endeavors to upscale channel count mainly by placing electrodes as close as possible, thus keeping physical expansion of the channel ensembles at the bottom (Pimenta et al., 2021; Steinmetz et al., 2021; Wang et al., 2021). The rationale behind spatial oversampling or spacing recording sites so tightly that SUAs and background activity are clearly separable is not only to boost recording quality and neuron discriminability (Diggelmann et al., 2018) but to assess the accuracy of a newly engineered spike sorting algorithm without knowing the ground truth data (Zhang and Constandinou, 2021a).

#### Microelectrode Arrays

Standing as the pedestal for BMIs, microelectrode arrays (MEAs) are capable of direct signal gain or transmission (Ravikumar, 2021). Knowing that the traditional single glass electrode, then later its wire version was quickly replaced by a bundle of four wires, it was quite reasonable that there was a demand-and finally solution for microelectromechanical systems (De Dorigo et al., 2018) and finally, densely packed MEAs with 8, 32, 4,096 or even 11,000, 65,536 recording sites. Another direction for sensor development is to create a flexible, mesh-like grid of surface electrodes, namely, electrocorticogram (ECoG) arrays; this too serves as a potential interface for invasive BMIs without direct damage to brain tissues. There are MEAs that enable recording on a single side of the shaft, such as Michigan arrays, while Utah arrays receive signals from the tip of silicon needles (Kim et al., 2019).

Objecting the vertical direction, Michigan probes are remarkably suitable for deep structure electrophysiological recordings. Their shaft length is usually between 2 and 15 mm (Choi et al., 2018; Ravikumar, 2021). Utah arrays, on the other hand, are 1.5-mm long, sharpened, and metal-layered silicon needles arranged in a matrix of 10 × 10 and are large enough to cover a 16-mm^2^ cortical, consequently apt, and approved by the United States Food and Drug Administration for clinical neurophysiology investigations (Saif-Ur-Rehman et al., 2019; Ravikumar, 2021; Sahasrabuddhe et al., 2021).

Apart from these traditional arrays, non-conventional array architectures purpose effectiveness and biocompatibility through multiple strategies. Prevention of array shielding may be reduced by multiplanar, robust arrays (Shin et al., 2017), but folding arrays in an origami style may also increase the surface from where recordings are gained (Goshi et al., 2018). Spatial resolution may also be improved by creating tubular recording devices (Wang et al., 2017). Conic recording structures help in chronic stabilization of recordings by enabling tissues to grow inside perforations (Hara et al., 2016); and ECoG-like design (Fu et al., 2017), extreme volume reduction (Ereifej et al., 2018), or even self-softening materials (Hess-Dunning and Tyler, 2018) aims to reduce adverse tissue reactions (Kim et al., 2019).

MEAs are excellent tools for long-term recordings, resulting in hundreds of relatively well-isolated single units (Chung et al., 2020). Nevertheless, we should not forget that detector/cell ratios that MEA arrays can provide are not always appropriate; hence, for tasks that require higher spatial resolution, probes may constitute a better choice (Negri et al., 2020).

#### Complementary Metal Oxide Semiconductors: CMOS Technologies

In the haste for superior recording instruments, lithographically printed and highly scalable probes turned out to be ideal candidates (Sahasrabuddhe et al., 2021). Being one of these, complementary metal oxide semiconductor (CMOS) applications amalgamated integrated circuits with recording electrodes, thus empowering probes with more compact input/output connections than ever (Hong and Lieber, 2019). CMOS probes maintain their compactness by local amplification and time-division multiplexing (Dimitriadis et al., 2018a) and support hardware acceleration processing by integration of application-specific integrated circuits or field programmable gate arrays at a very fair energy and space consumption rate (Schaffer et al., 2021); that is how they ensured the appearance of high-density microelectrode arrays (Dragas et al., 2017). An outstanding attempt are the Neuropixels probes: their silicon structure is made up of CMOS technology, with recording site numbers reaching up to 960 (Wang et al., 2021) or 5,120 (Steinmetz et al., 2021). Similarly grandiose projects made evidence that these types of probes are worth “scaling up” (Tsai et al., 2017; Sahasrabuddhe et al., 2021), reducing even more their inter-electrode spacing in Fiáth et al. (2019b) using them intracellularly (Abbott et al., 2020) or for the sake of neuromorphic computational paradigm (Milo et al., 2020).

### Raw Output Data

The plethora of neural sensors does not entitle us to choose the recording parameters further discussed in a rather oblivious manner; on the contrary, we should strive to precisely select our targets within brain structures and their specific physiological conditions for data acquisition, as quality of spike sorting does heavily lean on this step (Hildebrandt et al., 2017). Considering technological factors such as sampling frequency (Irwin et al., 2016), referencing procedures (Jurczynski et al., 2021) and recording data that might be subdivided later (Hassan et al., 2021) are all influencing quality and computational cost. We should also take into account that not every channel would provide the highest quality signal possible, since electrodes detached from their amplifier may broadcast their data intermittently or simply get distorted by noise. For this case, real-time rejection of corrupted channels would be favorable (Swindale and Spacek, 2016; Li et al., 2020a).

#### Need for Compression

If we record with 1,000 channels at a sampling frequency of 40 kHz, an hour is just enough to produce 30 GB of electrophysiological data (Hadianpour et al., 2021); furthermore, a Neuropixels probe of 384 channels generates 90 GBs when sampling is set to 30 kHz. Unless virtually infinite storage capacity is in our hands, compression is what we should make use of. A more compact dataset is not just efficiently, but also speeds up the computational process (Rokai et al., 2021). Several methods have been described for data reduction up to a four-fold rate, including pure compression (Pagin and Ortmanns, 2018), thresholded signal transmission (Irwin et al., 2016), or the lately introduced on-chip spike sorting procedures (Saeed et al., 2017; Xu et al., 2019). Local data reduction may enable wireless broadcasting (Schiavone et al., 2020; Sahasrabuddhe et al., 2021; Voitiuk et al., 2021) but cannot handle massive multiplexing (Muratore et al., 2019); thus, large-enough on-chip memory is an absolute prerequisite (Yu et al., 2011): for instance, operating with 128 channels, with each of them generating 32.5 samples per second, would have a memory requirement of 768 to even 2,400 kbits (Park et al., 2017). Such a chip could be just robust enough for deep learning applications, namely, compressing inputs into an output that can be deconvolved on the receiver side (Wu et al., 2018b). Instead of transmitting each recorded sample, the mean difference between every two of them, delta compression, seems a reasonable routine (Mukhopadhyay et al., 2018; Chou et al., 2021; Pagin, 2021), although compressed sensing techniques can render data even more compact (Xiong et al., 2018).

Sensor types can play an additional role in compression. Metal oxide memristive integrative sensors record and compress information in parallel (Gupta et al., 2016), while neuromorphic sensors are capable of event-driven recording and transmission, therefore improving temporal precision and reducing power consumption and data bandwidth (Liu et al., 2018; Soleymankhani and Shalchyan, 2021).

#### Digitalization of Neural Data

To be computationally analyzed, analog signals must be digitized, and analog to digital converters (ADCs) are just meant to solve the task. Various studies reported that for optimal spike sorting conditions, ADC resolution must be at least 7–8 bits (Zamani and Demosthenous, 2015; Liu et al., 2017; Pagin and Ortmanns, 2017). Moreover, logarithmic ADCs, as opposed to their linear counterparts, take advantage of small signals and more distributed dynamic range, just how neural recordings are designed to stand out.

## The Common Spike Sorting Procedure

After data acquisition and its conversion to digital signal, the search and contextualization of extracellular action potentials follows. This mining-and-meaning procedure has been coined spike sorting and subdivided into a changing number of tasks, like waveform identification, feature extraction and low-dimensional re-representation, and, finally, projection-based group formation (Fournier et al., 2016). In different stages of spike sorting, we could refer to preprocessing (cleaning) and processing-per-se practices, but it is quite reasonable that for practical and computational reasons, even major steps tend to interweave.

### Filters and Detectors

Prior to action potential detection, it is worth considering a filtering stage, as lower frequency local field potentials, mostly defined as frequencies below 300–500 Hz, may encumber further analyses (Issar et al., 2020). By this step, the quality of spikes should also enhance; hence, filters behave as balancing factors between incorrectly detected or discarded events even without previous thresholding (Zhang and Constandinou, 2021b). Take for example plain bandpass filtering (e.g., causal infinite impulse response filters), with the advantage of amplitude threshold detection: as feasible as it is, one should also deal with avoiding secondary phase distortion (Schaffer et al., 2021). Another yet computationally expensive non-linear filtering option is wavelet denoising, and the Haar mother wavelet is especially useful when background noise is unlike Gaussian distribution (Barabino et al., 2017; Baldazzi et al., 2020; Pakman et al., 2020). Statistical filtering is based on certain calculated parameters, like average absolute values or standard deviation of sample waveforms (Toosi et al., 2020), while reverse filtering ensures noise diminution by waveform encoding and restoration (Mizuhiki et al., 2020). If abrupt changes in particular data are suspected, particle filtering may confidently detect them, along with accepting the burden of greedy computational needs (Hu et al., 2018). Artifacts occur not just because of imperfect signal filtering but also human-induced signs like stimulation artifacts may contaminate data, and as these objects are highly structured artifacts, statistical filtering may circumvent this source of bias (Mena et al., 2017; Toosi et al., 2020). Despite most algorithms striding to suppress noise, some of them suggest highly contaminated snippet exclusion (Evangelou, 2020). At first sight paradoxically, introducing certain artifacts in the pre-emphasis (Ravikumar, 2021) with an optimum flicker noise intensity called stochastic resonance, signal detection rates significantly improve (Güngör and Töreyin, 2020; Güngör et al., 2021).

As it can be seen, filtering and spike detection are the lead-in operations in spike sorting; therefore, the quality of feature extraction and clustering is greatly impacted by detection algorithm performance, but even if data have been vigorously curated, spotting spike candidates remains a challenge (Okkesim et al., 2021). Filters may be an excellent support for threshold crossing event detection algorithms (Yang et al., 2017; Saggese et al., 2021), although more complicated methods, such as correlation-based detection, wavelet decomposition (Gao et al., 2018), Bayesian shrinkage methods (Sousa et al., 2021), and Teager or smoothed non-linear energy operators may also profit from them (Pagin and Ortmanns, 2017; Tambaro et al., 2020). Once noise level is estimated, amplitude threshold value can be set to a proper value (Barabino et al., 2017), although dynamic changes in noise variance are principally neglected (Toosi et al., 2020), but where is the optimum for picking a threshold? By employing a three-to-five standard deviation threshold, most authors agree that spike prominence is correctly estimated (Laboy-Juárez et al., 2019), while others focus on loss minimization and push threshold values lower (Bigelow and Malone, 2021; Chou et al., 2021). Instead of declaring signal standard deviation as the event detection threshold, it may be reasonable to surge robustness against alternating firing rates and calculate with median values (by recognizing that high amplitudes or spiking activity could only represent a small fraction of the recorded data) (Pregowska et al., 2019). Therefore, an automatically set threshold value could be determined as follows (Quian and Nadasdy, 2004):


(1) $$\begin{array}{r} \sigma_{n}=median\left\{\frac{\left|x\right|}{0.6745}\;\right\}, \end{array}$$

where x stands for the filtered signal. Evaluated at $\frac{3}{4}$, the approximation constant is the inverse of the standard normal distribution function, and threshold may be from a two- to four-fold value of σ_n_ (Quian and Nadasdy, 2004).

Besides threshold crossing, plenty of algorithms enable action potential detection. Smoothed or common non-linear energy operators may be capable of sub-millisecond on-chip spike detection (Malik et al., 2016; Schaffer et al., 2017; Tambaro et al., 2021). Signal-to-noise ratio can be further augmented by amplitude-slope operators (Zhang and Constandinou, 2021b). By applying a Teager energy operator-detector on data, even higher noise levels are well-tolerated; thus, filtering stages may be skipped (Lieb et al., 2017). Another noise-resilient approach represents fractal analysis of neural recordings, and after concluding that segments containing spikes have inferior dimensionality compared to noise, spike detection can be achieved (Salmasi et al., 2016). As it can be candidly imagined, an action potential and its propagation in an extracellular space would not let the entropy content of the temporal dimension unchanged, so calculating it with a sliding window method proved to detect spikes with greater specificity (Farashi, 2018). Combined methods that filter and set threshold parallelly, with adjustable weights depending on the source, are signal-to-noise ratio optimal filters and proposed to reduce computational complexity and upgrade discriminating capability (Wouters and Kloosterman, 2019). As a next chapter in filtering and detection paradigms, neural networks with barely one hidden layer can fulfill the tasks of preprocessing and event detection (Issar et al., 2020).

### Alignment

After successful data filtering and detection of action potentials, spike characteristics should also be explored and mapped. Before the very solution, which is feature extraction, it is reasonable to line up spikes in a way that may facilitate further processing and eventual visualization. This can be rendered by “binning” all spikes into a fixed length window, and then aligning them such that each spike has its temporal reference point, for instance maximum value or slope (Metcalfe et al., 2017; Valencia and Alimohammad, 2021). This method is uncomplicated and vital when opting for clustering alternatives but may fail when noise corruption is elevated (Valencia and Alimohammad, 2019). Such issues may be circumvented by upsampling data and, therefore, performing super-resolution alignment (Lee et al., 2017).

### Feature Extraction

The main pillar for accurate signal decoding is inarguably finding distinctive features in spikes that practically reveal their source. This gain of waveform information is called feature extraction, where only the most critical elements, the so-called principal components, are retained for further assessment (Ravikumar, 2021), which means that later, dimensionality reduction also takes place (Mahallati et al., 2019). For maximizing spike sorting accuracy, it is reasonable to choose principal components wisely, preferably ones that are noise-independent (Soleymankhani and Shalchyan, 2021) and are distinctly discriminative (Lefebvre et al., 2016; Zamani et al., 2020b) but cheap at implementation (Zamani et al., 2020a); by this means, we can map neural data in an informative but lower dimensional space. There is also a difference between first (waveform amplitude) and second (slope of the waveform) principal components (Navratilova et al., 2016). Principal component analysis (PCA), as one of the most popular dimensionality reduction methods (Salmasi et al., 2016; Allen et al., 2018), constructs a matrix of the largest variation-containing orthogonal basis vectors in the feature space (Chen et al., 2021), but extensive computations and storage requirements are inevitable (Regalia et al., 2016; Yang et al., 2017).

The must for unsupervised analysis had urged to think forward PCA. As a result, independent component analysis (ICA) has been created, which, similarly to PCA, has also benefited from redundancy reduction, improved signal to noise ratio, and as a final implementation, fastICA version reduced computation time, therefore turning the initial method into a suitable option for high density channel recordings (Leibig et al., 2016). While appropriate for high-density MEAs, ICA presumes that the set of sources does not outweigh the number of recording channels, and consequently fails when tetrode or low-density neural recordings are analyzed (Buccino et al., 2018). ICA generates both temporal and spatial redundancy, an advantage that can be brilliantly exploited by deep learning: convolutive ICA methods, therefore, are ready to extract features and cluster them in an unsupervised fashion (Leibig et al., 2016).

There is another dissimilar branch of methods that prioritizes cutting back on hardware complexity and template matching (Laboy-Juárez et al., 2019) that is an increasingly popular alternative for clustering. Discrete derivatives (Zamani et al., 2018) or optimal wavelet transforms (Yang and Mason, 2017; Soleymankhani and Shalchyan, 2021), which are sub-band selective, can stand for filtering as well (Soleymankhani and Shalchyan, 2021), whereas zero crossing features (Oh et al., 2017) or first and second derivative spike features (Caro-Martín et al., 2018) are methods that can tackle this condition. These methods are concentrated on global features gripping waveform morphology similarities of action potentials, but local feature extraction, e.g., Laplacian eigenmaps, could constitute another strategy as well (Chah et al., 2011; Huang et al., 2021). Regardless of the choice of feature extraction algorithms, by the end of this step, a well-represented feature space should be received, mapping each spike snippet as part of a highly distinguished and densely populated area (Chung et al., 2017).

### Clustering: The Core of Spike Sorting

**Figure 2:**
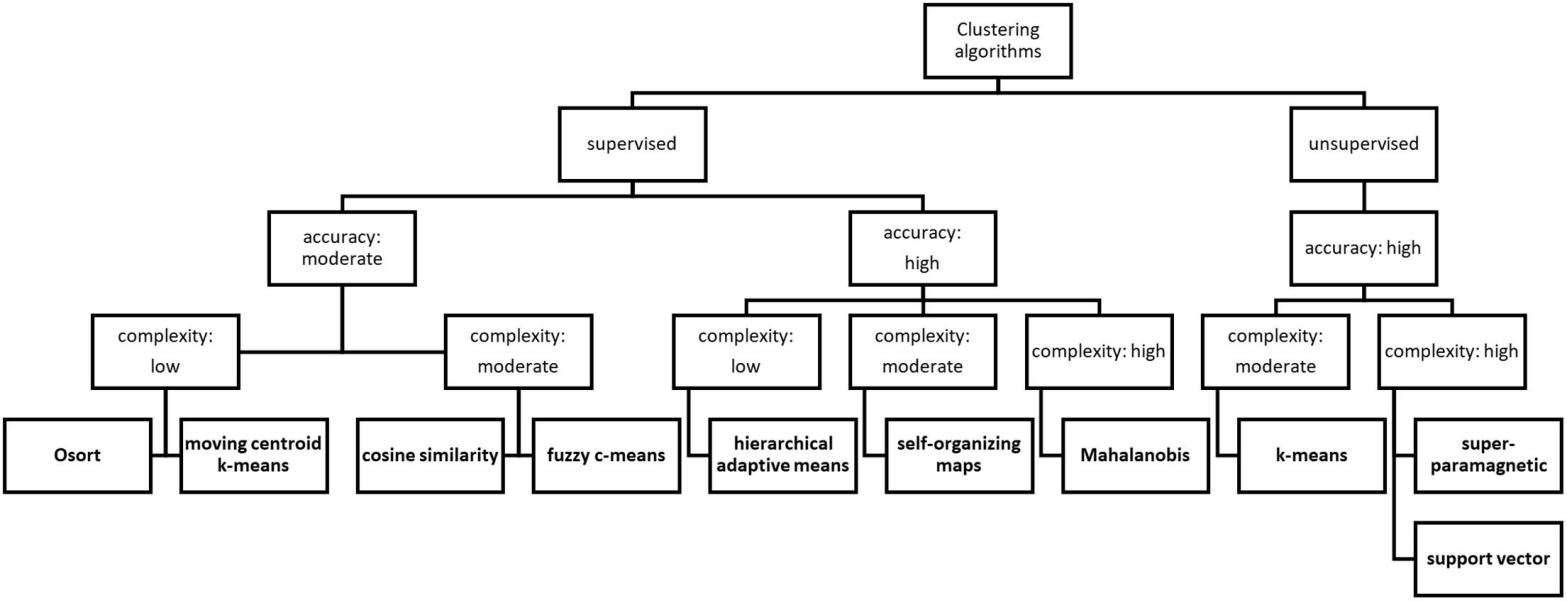
Feature classifications of the most widely used clustering algorithms. When choosing the ideal algorithm for a given dataset, it is straightforward to imagine a decision tree and consider which features are essential during the clustering process: supervised or unsupervised, and then the level of accuracy, even with acceptance of enormous computational costs.

The practice of categorizing spikes or their calculated features in such a way that their source neurons would be identical holds the name of clustering (Knieling et al., 2017). All the algorithms and technologies presented so far converge toward clustering, since the goal of decoding extracellular action potentials is acquired by this step. The ideal clustering algorithm runs real-time, implements sequential processing, it is fully unsupervised, but preferably as uncomplicated as clustering and parallel operations may be carried out on the recording device (Wood et al., 2004; Li et al., 2019; Toosi et al., 2020). For simplicity, clustering algorithms may be arranged into model- or non-model-based categories, admitting that even within these groups, algorithms highly differ from each other (Figure 2). This section intends to outline major clustering paradigm strategies without the ambition to compare all of them in terms of performance, execution speed, and other properties.

Model-based or simply probabilistic approaches lean on a source-dependent spike probability distribution provided by a generative model: Bayesian methods, expectation maximization procedures, or maximum likelihood estimations are typical instances of this class. These tactics are unusually resilient to noise associations, and over and above cluster visualization is facilitated (Mahallati et al., 2019). Modeling a mixture of drifting t-distributions enables differentiating overlapping clusters from heavy tails (Shan et al., 2017), while hidden Markov models have been successfully utilized during joint detection and sorting analyses (Li et al., 2019); however, the foremost strain of computational requirements is faintly resolved. The threat of cluster overseparation is also considerable, especially when non-Gaussian clusters, in fact, are assumed to represent Gaussian distribution (Keshtkaran and Yang, 2017; Rezaei et al., 2021).

Non-model-based methods, on the contrary, endeavor on classification tasks (Shan et al., 2017). With almost historical relevance, manual clustering is the most expository approach where obvious parameters like spike amplitude, duration, and channel location are applied as model substitutes (Sun et al., 2021). These methods have been gradually replaced by minimally supervised or supervision-free practices, one of them being k-means clustering, from the partitional subclass. Today, k-means dominates spike sorting protocols because of its lack of sophistication (Dallal et al., 2016; Fournier et al., 2016; Lefebvre et al., 2016; Park et al., 2020; Rácz et al., 2020), along with hierarchical techniques or graph-based, fuzzy logic, and density-, grid- and learning-based methods (Zhang et al., 2018; Veerabhadrappa et al., 2020). Hierarchical solutions are mainly represented by Euclidean distance-employing algorithms (Knieling et al., 2017), which are the base of optimal filter estimation methods, too (Hassan et al., 2020). Graph-based clustering has nearest neighbor interactions at the center, but spectral clustering (Huang et al., 2021) or super-paramagnetic clustering in the well-known wave_clus algorithm does also subscribe to this ground (Quian and Nadasdy, 2004). Fuzzy-C-means logic considers each action potential as a member of every cluster that has been delineated, and only their affinity degree makes decoding possible (Regalia et al., 2016). Density-based algorithms are the most analogous approaches with human clustering strategy, since they focus on agglomerated regions and their low-density belts in the feature space (Chung et al., 2017; Hilgen et al., 2017; Hennig et al., 2019). Learning-based clustering incorporates various means in the service of spike sorting, beginning from single-layer perceptrons to state-of-the-art spiking neural networks (Veerabhadrappa et al., 2020).

Making up our mind for a certain clustering approach does not automatically discard correction procedures or even melting it with another algorithm. The minor corrections imply Laplacian eigenmaps, which boost k-means clustering accuracy (Chah et al., 2011) or subset clustering together with pre-whitening for parameter-free spike clustering (Diggelmann et al., 2018). Consensus and ensemble clustering uses the variability of distinct clustering algorithms (Fournier et al., 2016; Vitale et al., 2019; Zhu et al., 2020); similar contracted measures are Euclidean distance of scatter-plotted features (Berjis and Al-sulaifanie, 2020), k-means clustering combined with mean-shift strategy [with the advantage of calculating the number of optimal clusters and analyzing similarity degrees (Negri et al., 2020)], and hierarchical agglomerative clustering (Cleaver-Stigum, 2021).

#### Automated Strategies

Gradual replacement of manual spike sorting by software techniques anticipated the rise of fully automated algorithms (Barnett et al., 2016). This particular aspect of spike sorting attracted so much attention in the past half decade, that this section is dedicated entirely to processes where tedious and time-intensive human interaction found its alternative.

Although most of the clustering algorithms presented previously are fully automated, many of them neglect aspects such as real-time application or opportunity to be uploaded on a chip that parallelly records and analyzes data. Neural network-based methods, however, are on the way to satisfy both criteria, with a promise of clinical applications (Radmanesh et al., 2021). But why do learning-based methods excel where human sorting efficiency is oftentimes inconsistent? Deep learning takes the advantage of non-linear relationship modeling, which means if associations between inputs and outputs are not straight-line, strategy finding patterns in these links might actually outperform algorithmic methods (Markanday et al., 2020; Guido, 2021).

Automated algorithms are characterized by an unsupervised strategy, although exceptions exist, such as supervised training of a neural network, to be followed later by unsupervised execution or selecting meaningful channels before the sorting process begins (Saif-Ur-Rehman et al., 2021). It must be emphasized, though, that most learning-based algorithms perform a plainer form of spike sorting, namely, classification depending on what has been learned during the paramount process of training (Luan et al., 2018). Classification may be a clever option when runtime drop is a priority instead of near-maximum accuracy (Valencia and Alimohammad, 2019), thus enabling online sorting on a general-purpose computer or a chip with which neural data have been acquired (Schaffer et al., 2021). Therefore, to achieve storage reduction, neural networks may be shrunk to three layers of artificial neurons, where additional attention elements complete the network (Bernert and Yvert, 2019). Sequentially constructed algorithms, such as those building upon multiple basic dense layers (Mahallati et al., 2019; Yeganegi et al., 2020) and convolutional (Li et al., 2020b) and recurrent layers (Rácz et al., 2020) require an expansive repository, although by weights' and activation functions' binarization, complexity may be cut back (Valencia and Alimohammad, 2021), or parallelization by graphical processing units may take place (Tam and Yang, 2018). These layers may be constructed in different ways, mainly in order to mitigate or abandon the need for hand-labeled neural data throughout training: autoencoders (Weiss, 2019; Radmanesh et al., 2021; Rokai et al., 2021) or networks generated by adversarial (Wu et al., 2019; Ciecierski, 2020) or reinforcement learning paradigms (Salman et al., 2018; Moghaddasi et al., 2020) have successfully clustered features originating from noisiest datasets. Likewise, a more sophisticated learning-based method may even incorporate multiple steps of spike sorting, resolving detection, feature extraction, and clustering as a close-packed solution (Eom et al., 2021; Rokai et al., 2021), although manual curation is advisable (Horváth et al., 2021).

Learning-based methods in the pay of automated spike sorting benefit a lot from additional remarks and optimization strategies (Table 1). If artificial neural networks perpetrate clustering, the optimal number of clusters may be estimated by Gap statistics (Tariq et al., 2019), and a method called Heuristic Spike Sorting Tuner even helps in selecting spatial or temporal features that ensure precise clustering (Bjånes et al., 2020). Regarding ideal input dimensionality, that is to say the number of specific features under analysis, studying four features are mostly sufficient for clustering (Hilgen et al., 2017).

**Table 1:** Presentation of the most used learning-based methods.

**Learning based method**	**Particularities**	**References**
MLP—multilayer perceptron	Non-linearly activating, fully connected nodes arranged in three or more layers	Park et al., 2020; Zamani et al., 2020b
CNN—convolutional neural network	Regularized MLP: finding hierarchies in data by sliding along convolutional kernels on the input matrix	Lee et al., 2017; Li et al., 2020b
RNN—recurrent neural network	Backpropagation of certain node outputs to previous layers for finetuning node values	Rácz et al., 2020
AE—autoencoder	Two-pillar architecture with non-recurrent neural network: 1. encoder encrypting the input; 2. decoder reconstructing the original input, based on the output of the encoder	Weiss, 2019; Eom et al., 2021; Rokai et al., 2021
GAN—generative adversarial network	Two-pillar architecture: 1. generative network creating samples for the evaluation performed by the, 2. discriminative network	Wu et al., 2019; Ciecierski, 2020
RL—reinforcement learning agent	Learning process based on maximizing reward after the action correctly executed	Salman et al., 2018; Moghaddasi et al., 2020

*It is worthwhile to stress that the first three learning-based methods are the fundamental structures of modern artificial neural networks; moreover they may serve as building blocks when constructing the latter three*.

**Figure 3:**
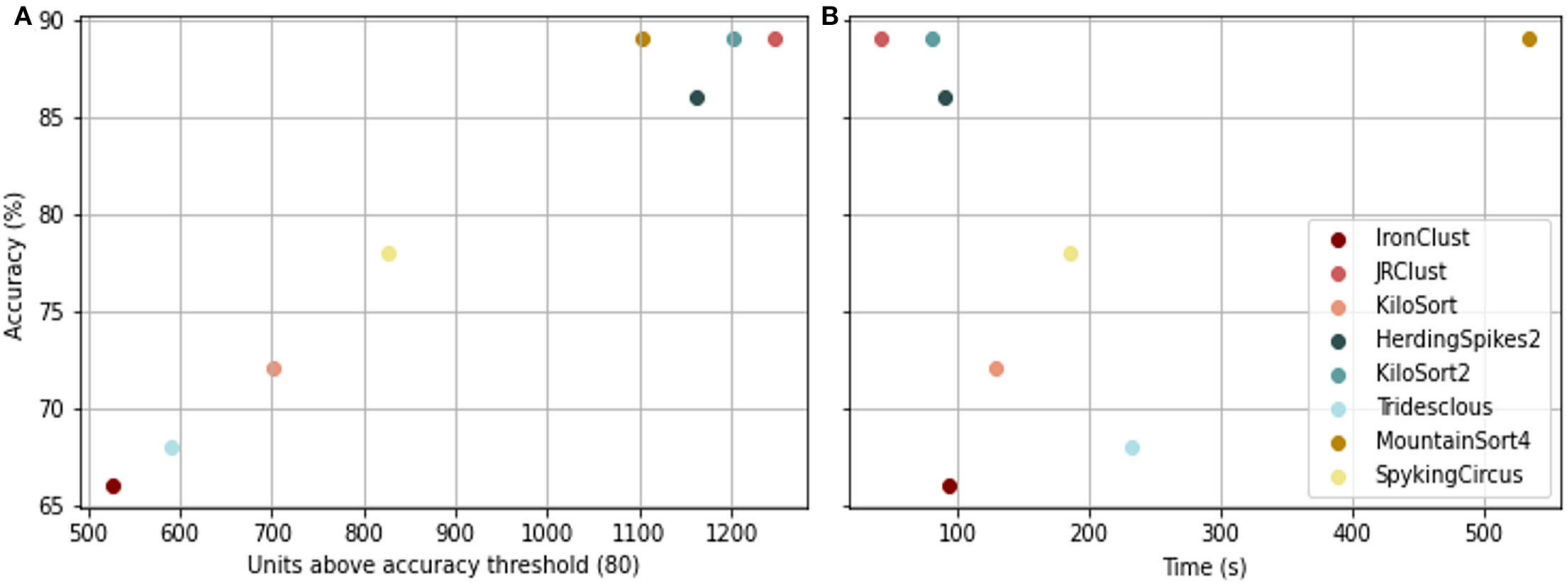
**(A)** Algorithm accuracy vs. the number of units found above the accuracy threshold of 80%. **(B)** Algorithm accuracy vs. time needed for computation. Algorithms were tested on the Hybrid_Janelia dataset, with a minimum SNR of 10^1^.

**Figure 4:**
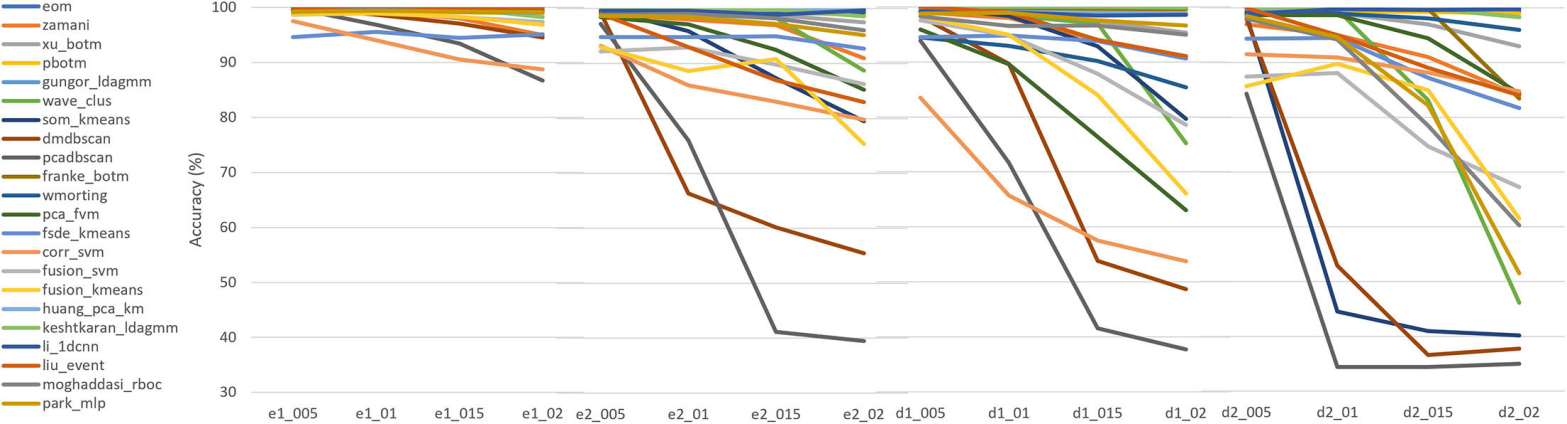
Comparison of clustering algorithms based on their accuracy achieved in the wave_clus dataset. We selected novel state-of-the-art algorithms that cannot yet be evaluated through the SpikeForest framework, but their performance in the wave_clus dataset has been made available with their publication. Generally, 16 samples of this dataset are applied during validation and divided into the subgroups “easy1,” “easy2,” “difficult1,” and “difficult2,” which appear in our x axis as “e1,” “e2,” “d1,” and “d2,” respectively. The values after the underscore reveals the noise content of each simulation, i.e., _005 means a 5% noise contamination, whereas _01 10, _015 15, and _02 20% sequentially.

Even with greatest circumspection during spike sorting, clustering quality must be inspected, and to solve the needs, ground truth-containing datasets have been created. These data entail a ground for fair comparison between spike sorting algorithms in terms of accuracy, speed, and memory requirements (Figures 3, 4).

#### Alternatives for Clustering

Clustering banks on feature space construction, which means that a two-step process may hide unanticipated problems. With this point of view, reducing the number of steps to 1 and introducing the concept of template matching hold great promises (Yang et al., 2017), especially when templates are interpreted in a Bayesian context (Franke et al., 2015). However, running templates through whole target signals in search for best-matching units is quite a chronophagous routine and remarkable when objected neurons are in abundance (Chen et al., 2021). As it could be speculated, these approaches are worthwhile when neural recordings are less compound, namely, electroneurogram/electromyogram decoding may be executed with this type of pattern recognition (Noce et al., 2019). By repeating spike sorting on the same data, normalized template matching methods guarantee an additional 40–70% detection of spikes; however, computational costs should also be a concern (Laboy-Juárez et al., 2019).

### On-Chip Spike Sorting

Spikes may even be decoded with a recording device, and power consumption; data quantity will also benefit from it (Liu et al., 2018). Contemporary tools to fulfill this task are mainly based on field-programmable gate arrays or application-specific integrated circuits (Barnett et al., 2016). Nevertheless, microcontroller units (Schiavone et al., 2020) and system-on-a chip devices (Liu et al., 2017) are increasingly popular. These chips, however, must be trained in preceding spike sorting procedure for successive fine tuning and accurate execution of functions (Zeinolabedin et al., 2016; Saeed et al., 2017). This approach is also vital in the field of neuromorphic computation, ignited by very large-scale integration technologies (Mukhopadhyay et al., 2018); there has recently been an implementation for 65,536 simultaneously recording and stimulating electrodes (Tsai et al., 2017). Similarly, extensive neural recordings are preferentially processed in batches and then subdivided into bins given the resistive state that calls for proper noise estimation (Gupta et al., 2019).

Clustering itself can be any algorithm from those introduced in the general clustering section, but it is worth considering the computational bottleneck of recording front ends. As it could be seen, most non-model based clustering algorithms are intended to capture specific geometric features; therefore, finding the most prominent ones could also cut back on the number of features under analysis (Shaeri and Sodagar, 2020). Event-trace, template-value differences add a temporal dimension to the template matching procedure, so they can further reduce the number of comparison units (Haessig et al., 2020). Learning-based methods may also control this circumstance at a superior level; hence, it is sufficient to upload a ready-to-use, modest as possible pretrained artificial neural network (Valencia et al., 2019). In this subject, hardware-embedded spiking neural networks are the greatest novelty, mostly owing to their feasible adaptation to data recorded on the fly (Werner et al., 2016).

### Toolboxes and Software Suites for Spike Sorting

The ever-increasing need for spike sorting has led to a wide range of open-source electrophysiological platforms, frameworks, and software (Pachitariu et al., 2016). Any related project is either aimed to bring spike sorting closer to a user who has little knowledge of the procedure or provide a space for methodology comparison and dataset generation. It is also required to support a wide range of data formats, as well as oscillating quality and length of the recordings (Swindale et al., 2017). Here, we briefly present novel toolboxes from the past 5 years that can be applied straightforward even by non-expert users.

For data acquisition, the Parallel Ultra-Low-Power (PULP) platform merges the process of data acquisition and single unit detection under a single benchwork (Schiavone et al., 2020). Another software, Neural Parallel Engine, is useful when the execution speed of spike sorting algorithms is crucial, since it enables highly parallelized computational process through graphical processing units (Tam and Yang, 2018). Neurophysiological data can be curated and further analyzed with Phy^2^, a graphical user interface operating with Python. Another manual curation-supporting graphical user interface is based on t-student stochastic neighbor embedding (Dimitriadis et al., 2018b).

Python frameworks have also been created for complete spike sorting procedures such as OpenElectrophy (Rosenberg and Horn, 2016), herding_spikes (Muthmann et al., 2015), NeoAnalysis (Zhang et al., 2017), tridesclous^3^, and spyke^4^. All of them support multichannel architectures. At the center of MountainSort, a density-based clustering approach stands, namely, ISO-SPLIT; this suite is also open-source (Chung et al., 2017).

SpikeInterface is a framework that not only offers algorithms for spike sorting, but most recent sorters can be used interchangeably (Buccino et al., 2020). Similarly, Spikeforest is also available for a wide range of sorting approaches; moreover, their comparison has never been easier because of its intuitive graphical user interface (Magland et al., 2020). Spikeforest, which may be embedded into the SpikeInterface environment, evaluates algorithms automatically and systematically based on some of the most well-known datasets supplied with ground truth. The Spike-Sorting Evaluation Initiative and the 1st INCF Workshop on Validation of Analysis Methods (Denker et al., 2012) have pushed the spike sorting community toward sharing essential data for algorithm evaluation, but cloud computing as a helping feature is also considered (Mahmud and Vassanelli, 2019).

When searching for unified computational for a more specific use, P-sort is a unique pipeline and software, destined to sort cerebellar single unit activities (Sedaghat-Nejad et al., 2021). CellExplorer suits for single-neuron characterization and signal visualization are for those who are confident with MATLAB (Petersen et al., 2020), whereas Big Neuronal Data Format (BNDF) emphasizes large-scale data processing and reducing overall runtime (Hadianpour et al., 2021). Combinato, on the other hand, is written in C/C++ and enables dealing with long-term, noisy recordings mostly in an unsupervised fashion (Knieling et al., 2016). For algorithm validation, SHYBRID is a surrogate platform when ground truth information is expected, offering hybrid data as an evaluation tool (Wouters et al., 2021).

**Figure 5:**
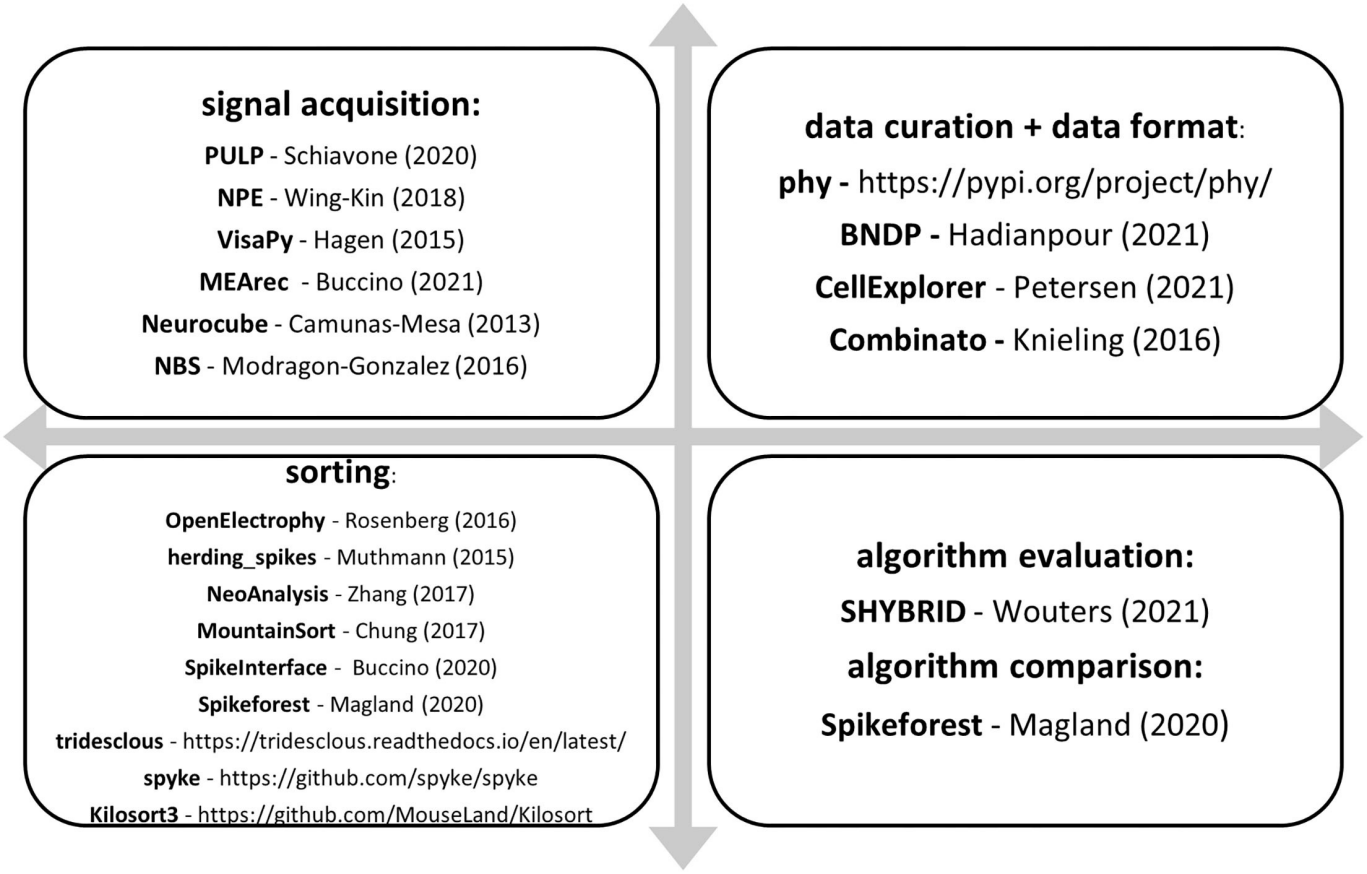
Enlisting signal processing toolboxes based on their most useful functions. “Signal acquisition” box: a wide variety of toolboxes treating difficulties of the recording or signal generation process, most of them compressing or prefiltering data for further steps. “Data curation + data format” box: software indicated for preprocessing previously recorded signals, most of them recommended even in the case of manual spike sorting. The “sorting” box lists the most used and trusted spike sorting algorithms or algorithm collections that can be applied to various datasets. Finally, the “algorithm evaluation” and “algorithm comparison” box offers help when a custom algorithm needs validation or measuring against the algorithms itemized in the “sorting” box.

Another road to unburdening spike sorting is to simulate datasets in which algorithms can be trained, validated, and tested. ViSAPy (Hagen et al., 2015) and MEArec (Buccino and Einevoll, 2021) are Python-based, whereas Neurocube (Camuñas-Mesa and Quiroga, 2013), Neural Benchmark Simulator (Mondragón-González and Burguière, 2017), and SHYBRID (Wouters et al., 2021) are MATLAB-implemented frameworks (Figure 5).

## Arising Challenges

There are several factors that may emerge during data collection, and despite being often predictable, they may complicate spike sorting. In the search for accurate and real-time functioning, computationally efficient algorithms, difficulties such as involuntary drifts, temporally coincident, overlapping, spikes, or even obstacles given by the ever-increasing recording capacity are generated. Disregarding energy consumption especially when implantable devices are considered can lead to obstacles in practice (Mukhopadhyay et al., 2018; Okkesim et al., 2021). Last but not least, we should also engage in assumptions that are made before recording, since electromagnetic field theory presumes extracellular space isotropy and homogeneity; however, e.g., privileged cell orientations and the bare existence of neural probes in a tissue render simple models inaccurate (Buccino et al., 2019). These notions are generally applicable within the gray matter, since one can easily admit radical differences in white matter signal propagation.

### High Channel Counts

By increasing channel counts and covered brain areas, we get an insight into more neurons' activities in parallel. This means that statistical models based on such recordings would also hold a promise for superior neural population activity interpretation (Hurwitz et al., 2021). Nevertheless, computational costs escalate almost exponentially with channel numbers, and the number of wires that send signals forward would also grow, except for methods employing controllable switches to attach recording sites with single wires (Lee et al., 2021). Increasing channel density and adopting a divide-and-conquer processing strategy (Chen et al., 2021) allows for effortless detection of duplicated or overlapping spikes (Larionov et al., 2019; Chou et al., 2021; Dehnen et al., 2021) and provides more detailed spatial information on action potential sources (Rácz et al., 2020). Meanwhile, by increasing the number of spikes sorted out, false positive or negative detections become an imaginable source of errors: a small quantity of mistakenly identified spikes perturb firing rate or interspike interval values (Chiarion and Mesin, 2021).

### The Drifting Dilemma

For a short time, almost every detected spike waveform keeps its original shape, which is the pseudo-stationary stage of the recording, and any occurring signal variability is mainly given by Gaussian-distributed noise (Yu et al., 2011). On the other hand, *in vivo* and sometimes *in vitro* setups suffer from small changes in electrode positions, even from the beginning of recordings, when, e.g., perceptible discrete brain sample dislocations that may impact initially recognized waveforms to an extent that clusters may split or even intercept each other and form new but misclassified groups of action potentials (Gong et al., 2016; Harris et al., 2016; Chaure et al., 2018). The most plausible solution for probe drift is to track and update primordial neuron templates (Lee et al., 2017) or handle data as independent batches, and then ultimately merge alike-looking clusters, but considering spikes with a mixture of drifting t-distribution character can also eliminate drift-suggesting heavier tails of clusters on data representation (Shan et al., 2017). Spike amplitude change may also be estimated with recursive least-squares, but its utility is mostly demonstrated by low-count channel recordings (Davey et al., 2020).

### Overlapping Spikes

Overlapping spikes frequently cause a problem for spike sorting algorithms: when different neurons fire in such a restrained time window that their waveforms overlay (Rey et al., 2015), the features of their subcomponents extracted for clustering cannot be applied. It is reasonable and right to suspect that the extent of overlap defines the trouble of accurate classification; yet quite paradoxically, evidence shows that firing rate and spike-train correlation levels do not infer with algorithm performance change (Garcia et al., 2021). If data suggest that these events, by incidence, are sufficiently low, simply censoring spikes with double peaks may overcome the problem, and for detections that represent only a mild overlap, deconvolution can be employed (Li et al., 2020b). Others emphasize the ubiquity of overlapping spikes especially when recording with high-density channel count probes and urge for specific algorithms committed to defeat misclustering. A straightforward approach suggests resolving the overlap in the feature space by treating problematic spikes' feature vectors as linear superpositions (Wouters et al., 2020), enhanced by one-hot encoding (Wouters, 2020), or quite the contrary, by fusing features in behalf of dimension number and missing information reduction (Li et al., 2018), while others suggest adding an extra step of combining pair-wise action potential waveform templates at various time shifts for refining sorting accuracy as much as 30% (Mokri et al., 2017). Additional alternative strategies are biogeography-based optimization (Chiarion and Mesin, 2021), using blind source separation methods (Leibig et al., 2016) or close examination of each spike cluster's center, which is equally close to another two midpoints (Wouters and Kloosterman, 2021), by automated template merging (Chen et al., 2021). Sparse representation or compressive sensing of neural data performs peculiarly well when spike waveforms are alike; therefore, overlapping spikes can be resolved by this means (Wu et al., 2018a; Huang et al., 2020). Wavelet Packets Decomposition and Mutual Information (WM sorting) is a clustering algorithm specially designed for overlapping spikes outperforming most of the methods presented here; nevertheless, its computational intensity generates doubts about real-time applications (Huang et al., 2019).

### Neural Bursts

Noticing in a recording channel a set of action potentials, with short, ~3–5-ms interspike intervals, mostly similar in shape but decreasing in amplitude, conventionally means there is a neural burst or “complex spike” (Rey et al., 2015; Evangelou, 2020). Emitted usually by pyramidal cells, in the standard signal generator circuit and by interneurons in deeper structures, the latter ones act by suppression (Gainutdinov, 2021). But why do they constitute a problem when spike sorting is at stake? First, decreasing amplitudes may hamper detection; moreover, this decrescendo may be the source of falsely created separate clusters; second, they can mimic other problem sources: complex waveforms may originate from overlapping spikes (Rácz et al., 2020), and diminishing may be due to electrode drifting. Bursting neurons can be handled by assigning them a burst index (Sun et al., 2021) or a special spike label (Kapucu et al., 2016), or by simply decomposing a spike train and its successors into a parent wave (Chung et al., 2017).

### Long-Term Recordings

The ability to achieve recordings from freely moving animals with a promise of proper neural decoding over months, hopefully with the same device, is the holy grail of spike sorting (Shan et al., 2017). Long-term recordings, therefore, comprise all arising challenges that could be met throughout spike sorting, but the main problem is represented by unit instability, when new-found spike waveforms replace former ones; hence, most template matching strategies fail (Toosi et al., 2020). Unit firing rate variations, neural plasticity, or loss of recorded neurons in the long run may also influence sorting accuracy (Okun et al., 2016; Xiao et al., 2019; Vasileva and Bondar, 2021), although large-scale, multisite platforms may reconcile even these obstacles (Chung et al., 2020; Vasileva and Bondar, 2021). In the long run, neural sensors may determine leptomeningeal proliferation and fibrosis or even foreign body reaction, all these possibly leading to device encapsulation, further deteriorating recording quality (Szymanski et al., 2021).

### The “Dark Neuron” Problem: Scarcely Firing Cells

It has been widely debated why generally, a recorded neural activity is sparser compared to anatomy-based expectations (Ahmadpour et al., 2019), However, research may suggest that the “dark neuron” problem could be accounted to neural sensors, since their de facto electrode sensibility is usually lower than their anticipated value; therefore others caution on the non-negligible nature of subthreshold signaling and urge for pushing further technical limitations (Shmoel et al., 2016).

## Validation of Spike Sorting Algorithms

It is a common consensus that any novel algorithm must at least show a better performance than its predecessors; however, inventing custom metrics, for instance isolation, noise-overlap, or cluster signal-to-noise ratio, may bias performance evaluation (Chung et al., 2017). To prevent this situation, evaluation metrics should be kept as simple as possible, relying on accuracy, precision, recall, or F1 scores (Veerabhadrappa et al., 2020). However, profiting from external criteria indices, such as the Jaccard index, that can compare data clustering structures and internal criteria indices, e.g., the silhouette coefficient or Calinski-Harabasz index, which is also meant to predict the optimal number of clusters, can all be beneficial (Zhang et al., 2018; Toosi et al., 2020) (Table 2). Another heuristic alternative for clustering accuracy is to measure its stability, which means that performance is evaluated in terms of perturbation introduction to the testing dataset (Carlson and Carin, 2019). Besides algorithm validation, performance of neural sensors must also be characterized: additional optical imaging with high spatial-temporal resolution can settle both aspirations (Aqrawe et al., 2020).

**Table 2:** Formulas of the most common clustering metrics and indices.

Performance quantification: • relies on ground truth • based on confusion matrix results	External criteria indices • relies on ground truth • compares calculated labels with actual ground truth
$Accuracy=\frac{TP+TN}{TP+TN+FP+FN}$	$Jaccard\;index=\frac{\left|D_{ground\;}\cap \;D_{calc}\right|}{|D_{ground}\;\cup \;D_{calc|}}$
$Precision=\frac{TP}{TP+FP}$	Internal criteria indices • requires no ground truth • gives information about cluster compactness and separation
$Recall=\frac{TP}{TP+FN}$	$Silhouette\;index=\;\frac{d\left(x,\;\bar{n}\right)-d\left(x,\;\bar{s}\right)}{max\left(\bar{s},\;\bar{n}\right)}$
$F1\;score=2\times \frac{Precision\times Recall}{Precision+Recall}$	$Calinski-Harabasz\;index=\;\frac{tr\left(B_{k}\right)}{tr\left(W_{k}\right)}\times \frac{n_{D}-k}{k-1}$

*TP, true positive; TN, true negative; FP, false positive; FN, false negative; Dground, dataset of the ground truth labels; Dcalc, dataset of the calculated labels; x, given sample point; $\bar{n}$, mean of all sample points from the next nearest cluster; $\bar{s}$, mean of all sample points from the cluster to which x is assigned; k, number of clusters; tr(Bk), between-group dispersion matrix trace; tr(Wk), within-cluster dispersion matrix trace; and nD, cardinality of the dataset*.

### Ground Truth

Validation of spike sorting performance is almost inconceivable without ground truth. This concept is based on the *a priori* knowledge of an action potential source, more specifically, sensing when and which neuron in the neighborhood was active (Neto et al., 2016). Ground truth data generation involves human interaction to a certain extent, turning it to a supervised process (Wouters et al., 2021).

What can be done when “ground truthing” is not an option? The most straightforward and popular method is to generate synthetic data and, with these, access ground truth (Buccino and Einevoll, 2021). Hybrid ground truth is accessible by spatially oversampled data acquisition or synthesis (Pachitariu et al., 2016; Scholvin et al., 2016; Wouters et al., 2021), although enriching data with formerly isolated or artificial spikes is also an option, coined hybrid method (Yger et al., 2018). Some classical approaches include manual labeling or simultaneous recording of intra- and extracellular activity, with the latter providing ground truth regarding a single neuron (Fournier et al., 2016; Diggelmann et al., 2018; Abbott et al., 2020). Recently, an automatic patch clamp technique *in vivo* lightened the workload, but human intervention cannot yet be erased (Kodandaramaiah et al., 2016; Allen et al., 2018). Simultaneous juxta-and extracellular recordings are also applied instead of *in vivo* patch clamping (Hunt et al., 2019; Magland et al., 2020; Urai et al., 2021). An appealing alternative for compensation of ground truth is to follow over the long run extracellular action potential propagation alongside single axonal arbors and, therefore, assessing their correlation with relatively stable extracellular action potentials; empirical ground truth can be obtained (Tovar et al., 2018). A last compromise for lacking ground truth can stand in the application of the internal criteria indices presented previously (Zhang and Constandinou, 2021a). This choice may be shadowed by certain tacit assumptions of internal criteria applications, such as the Gaussian nature of noise distribution.

#### Simulated Datasets

For the past 15 years, reclining on the well-known wave_clus dataset (Quian and Nadasdy, 2004) has proved to be a reliable source for algorithm validation; moreover, its prevalence for this scope assured a feasible and fair comparison ground among clustering procedures. Analogous but less-known synthetic datasets also grant for correct validation (Rutishauser et al., 2006; Pedreira et al., 2012; Rossant et al., 2016). It is becoming increasingly popular to generate custom datasets, enabling to set various complexity levels or recording site geometries (Smith and Mtetwa, 2007; Camuñas-Mesa and Quiroga, 2013; Hagen et al., 2015; Mondragón-González and Burguière, 2017; Buccino and Einevoll, 2021), but when producing synthetic waveforms that ideally mirror recorded ones, one should also pay attention to cell morphology, membrane ionic channel configuration, and density (Tran et al., 2020). Paying attention to previously lesser-considered aspects like layer-in homogeneity and dependence of frequencies resulted in construction of trailblazing dataset-simulating environments (Gherardi and Toreyin, 2021) (Table 3).

**Table 3:** Most relevant and widely used synthetic datasets for spike sorting algorithm validation.

**References**	**Specifications**
Quian and Nadasdy (2004)	wave_clus dataset: activity of 3 simulated neurons over 4 difficulty levels. The noise level is well-defined in each case.
Rutishauser et al. (2006)	3 simulated datasets each containing 3 neurons with the following scopes: • 1st: parameter evaluation for the algorithm tested • 2nd: limits of detectability • 3rd: limits of discriminability.
Smith and Mtetwa (2007)	Biophysical model incorporating signals that closely mimic *in-vitro* single unit activities, adding non-Gaussian noise and featuring spatial configuration characteristics between neurons and recording electrodes.
Pedreira et al. (2012)	95 simulations containing from 2 to 20 neurons' action potentials. The background activity appears as multiunit activity of varying weighting.
Camuñas-Mesa and Quiroga (2013)	A tetrode simulation with neural and thermal noise incorporated, three-dimensional aspect enhanced by the “neurons inside a cube” concept.
Hagen et al. (2015)	VisaPy: *in vivo* mimicking of the multi-compartmental neuron model. A Python-based software is also available.
Rossant et al. (2016)	Klusta: test dataset available for the algorithm proposed in the same paper.
Tran et al. (2020)	NEURON-based model software employing morphological filter for signal accuracy.
Gherardi and Toreyin (2021)	NEURON-based model software with line-source approximation improvements.
Buccino and Einevoll (2021)	MEARec: signal generation considering problematic spike aspects, several electrode designs

*Note that while pioneering data have been constructed to test a certain algorithm; these days, data-synthesizing types of software prevail, with all their custom implementations*.

#### *In vivo* Datasets

Despite lacking ground truth information, *in vivo* recorded datasets are highly valuable, because all features that simulated datasets need to be fulfilled are implicitly present. To mention a few, for rat cortical recordings, there are 32/128 channel-polytrode (Neto et al., 2016), 128 (4 × 32) array (Horváth et al., 2021) and Neuropixels-patch clamp combined recordings (Marques-Smith et al., 2018) available. A dataset recorded by Utah arrays on non-human primates executing different tasks is also suitable for single-unit activity clustering (Brochier et al., 2018). Human data are regularly acquired from patients whose epileptic seizure onset zone is under investigation. Among these, amygdala neurons during visual/emotional stimulation (Fedele et al., 2021) or medial temporal lobe populations under verbal working memory tasks are recorded by intracranial EEG and technically validated later (Boran et al., 2020) (Table 4).

**Table 4:** Most recent *in vivo* datasets suitable for validation of spike sorting algorithms.

**References**	**Source**	**Recording device**	**State or task**	**Dataset**
Neto et al. (2016)	Rat cortex	32- or 128-channel polytrode	Under anesthesia	23 neurons with juxtacellular pipette + nearby electrode
Marques-Smith et al. (2018)	Rat cortex	Patch clamp next to Neuropixels probe	Under anesthesia	43 paired recordings
Boran et al. (2020)	Human medial temporal lobe	Depth electrode with 8 contacts	Working memory task	9 patients, 37 recording sessions
Brochier et al. (2018)	Macaque motor cortex	10-by-10 Utah arrays	Reach-and-grasp task	2 macaques, 93 and 156 single unit activities
Fedele et al. (2021)	Human amygdala	Depth electrode with 8 contacts	Various emotional situations	9 patients, 14 amygdala recordings
Horváth et al. (2021)	Rat neocortex	128-channel polytrode	Under anesthesia	20 rats, 109 recordings, 7,126 sorted spikes

*While there is a clear aspiration for acquiring continuous long-term recordings with as many channels as possible, and preferably although not necessarily from humans, all criteria cannot always be met. However, datasets focusing on just one of these aspects can reveal important strengths and weaknesses of a spike sorting algorithm under validation*.

## Discussion

### On the Necessity of Spike Sorting

Spike sorting is not an art for art's sake protocol, since its applications are visibly boosting contemporary neuroscience: it has become essential in extraction of individual neuronal activities from multi-electrode data, since each electrode reports the collective activity of multiple nearby neurons. But why is carrying out this task cardinal? The fact that neighboring neurons are not necessarily the nearest in connections, i.e., are activated by different pathways or stimuli (Rey et al., 2015) mainly owing to information processing and energy optimization through structural solutions (Pregowska et al., 2019) calls the promise of neural decoding. This concept, although not equal with spike sorting, relies heavily on it and undertakes the risk of bias and wrong intensity estimation generated by spike sorting (Shibue and Komaki, 2017). Spike sorting also incites statistical analyses, involving correlogram analysis, inter-spike intervals, or spike rates (Veerabhadrappa et al., 2020).

When we talk about spike sorting we classically imply manual sorting alongside automated methods (Dai et al., 2019). Given its time-consuming feature and potential for subjective bias (Febinger et al., 2018), manual sorting has been mostly overtaken by history; consequently, there is a sore need for fully automated algorithms (Chah et al., 2011). High-density microelectrode arrays foster progressing classification accuracies; however, computational capacity should also fall in line with recording performance (Chen et al., 2021). Several authors, most of them stressing the expenses of calculation, argue against the pertinence of spike sorting. As it may be expected, alternatives for spike sorting all have their strengths and limitations. According to those who subscribe to rather overtake spike sorting, when the sum (Li and Li, 2017) or moments (Sonia et al., 2014) of waveform features are calculated, spike sorting can be omitted for motor imagery task neural decoding; however, even these methods fall short of real-time reconstructions. Similarly, frequency spectrum maps together with temporal energy heatmaps can predict imaginary finger movements but in a well-defined force amplitude interval (Xu et al., 2020).

Spike sorting indeed does not have to be compulsory when population activity is targeted (Trautmann et al., 2019); therefore brain-computer interface systems that engage in multi-unit activity may settle for less complex preprocessing techniques. Such a method is “binning,” by which firing rates are estimated in a fixed time window (Ahmadi et al., 2020), and with marked point models (Yousefi et al., 2020) or interspike interval histograms combined with power spectrum density estimation, complete firing patterns can be investigated (Guo et al., 2020): these methods are advantageous when tuning curves of single neurons may be distributed bimodally, such as in the case of murine head direction cells (Liu and Lengyel, 2021). Whenever applying a clustering-free method, one should keep in mind that its goodness-of-fit evaluation may differ from mainstream clustering algorithms (Tao et al., 2018). Nonetheless, at the level of individual cells, properties cannot be completely evaluated with spike sorting algorithms (Rossi-Pool and Romo, 2019). To conclude, an eventual conflict between spike sorting methods or data should not discourage us from conducting spike sorting whenever there is a clear indication of employing it.

### Implementations of Spike Sorting

As the demand for spike sorting is clear-cut, we considered further stressing the relevance of these sets of algorithms by presenting research fields that greatly benefit from spike sorting. Spike sorting is mostly regarded as the essential step toward functional brain-machine interfaces and micro-electronic neural bridges (Huang et al., 2016), but its relevance is substantial in epilepsy research (Neumann et al., 2017; Richner et al., 2019) or the study of sleep (Kozák et al., 2020; Matsumoto, 2020). Besides common-term primary motor cortex single unit activity, clustering in areas where visual coding or multisensory integration takes place is an intuitive approach in vision and adaptive behavioral studies (Reber et al., 2019; Mizuhiki et al., 2020; Steinmetz, 2020). Cerebellar spikes should also be detected and clustered; nevertheless, the latter task is rendered more difficult by the intricate morphology of Purkinje cells (Markanday et al., 2020). Complex spikes can also be met in the thalamus, and spike sorting is just a way to learn about its electrophysiological properties (Pastor and Vega-Zelaya, 2020). In the subthalamic nucleus, clustering of spike trains may help to understand the pathophysiology of movement disorders (Kaku et al., 2019; Sukiban et al., 2019), while the basolateral amygdala or the hippocampus can offer ideas about general neural interactions (Hojjatinia et al., 2020; Oghazian et al., 2020). Correlations calculated after vigorous spike sorting in multichannel data gave rise to a promising neural encoding capacity hypothesis (Isbister et al., 2021) and neural populational activity dynamics (Theilman et al., 2021).

Furthermore, spike sorting may be just as pivotal outside of the brain. Remaining at the level of the central nervous system, superficial dorsal horn spinal cord neurons reveal much about spinal plasticity, and sorting their activities may ensure isolation of relevant populations (Smith et al., 2020). For peripheral nervous system recordings, discrimination and identification of APs cannot lack spike sorting either (Metcalfe et al., 2021). Spike sorting is required to isolate retinal ganglion cells based on their multiunit activity recordings (Tsai et al., 2017; Pérez-Ortega et al., 2021) and identify their electrical responses (Li et al., 2021), but non-neural tissues can also benefit from it by applying spike sorting algorithms, i.e., on pancreatic biosignals (Iniguez-Lomeli et al., 2021).

Spike sorting has its own advantages during *in vitro* experiments, too. Cerebral organoids, also known as Minibrains, and spike sorting together enable studying neuro/gliogenesis and connectivity (Govindan et al., 2021), while spike sorting in its intracellular variations can elucidate synaptic plasticity mechanisms (Ghanbari et al., 2017). Recently, spike sorting proved to be inescapable when performing optogenetic stimulation on MEA-supported brain slices (Sacher et al., 2021).

Up to this point, we presented spike sorting as a complete procedure that seeks to assign a signal to a particular source, but regarding it as a piece of puzzle toward another problem solution is also valid. Spike sorting can be embedded in synaptic connectivity estimation algorithms, thus helping construct neuronal circuit diagrams (Endo et al., 2021). By combining spike sorting with phasic unit selection, we can recognize firing patterns in structures that have timekeeping properties (Chrobok et al., 2021).

### Past Conclusions, Current Improvements, and Future Ideas

Our study intended to outline some of the most relevant issues that shape current spike sorting trends. Despite the endless attempts and various strategies, obvious spike sorting standards and algorithm comparison methods are still deficient, but any effort toward unified spike sorting frameworks and accuracy quantification metrics should be cheered (Rey et al., 2015; Smith et al., 2020). In spite of closely focusing on a set of algorithms that ascribe a well-defined signal to its supposed emitter, we should not miss the bigger picture either and, therefore, concentrate on the non-independent nature of neural activities (Urai et al., 2021). In the search for the gold standard algorithm, we should also leave room for neural network-based or custom-tailored solutions too, without which we cannot excel when miscellaneous temporal or spatial particularities are present (Vu et al., 2018; Sedaghat-Nejad et al., 2021). The authors of this article believe that fostering the concept of incremental learning in spike detection and clustering methods could offer the panacea for most difficulties arising during long-term recording analysis, since adaptation to alternating circumstances would not demand repeated training on previous samples but focus on the gradually growing set of data without having to worry for memory restrictions.

Another aspect that opens new perspectives is the myriad of neural sensors at our fingertips. As it can now be anticipated, CMOS technologies could fulfill the requirements for implantable long-term sensors, as their ultra-low power consumption, neuromorphic design, and, lately, their capability to self-repair should be exploited (Rahiminejad et al., 2021). Even though a great variety of recording devices provides us with data of ever-improving signal-to-noise ratios and of diminishing invasiveness, there are fundamental questions about extracellular action potentials that are, so far, unanswered. In particular, a probe's interference with the adjacent neural tissue, eventual chance of bias for certain neuron subclasses, cross-compliance between neuron types, and extracellular signatures are hotly debated topics (Neto et al., 2016). None of these subjects are self-standing, since contemplating about the limitations of recording facilities could also possibly bring about the advent of better-built BMI systems.

## Author Contributions

RB conceived the study, conducted relevant literature search, and wrote the first draft. JR and GM contributed in and supervised the drafting process. JR, GM, RF, IU, and DM revised and suggested modifications on the manuscript. RF provided data for figure generation. JR helped in data formatting. All the authors contributed, read the manuscript, and approved the submitted version.

## Funding

Project no. FK132823 has been implemented with the support provided by the Ministry of Innovation and Technology of Hungary from the National Research, Development and Innovation Fund, financed under the FK_19 funding scheme. This research was also funded by the Hungarian Brain Research Program (2017_1.2.1-NKP-2017-00002) and the TUDFO/51757-1/2019-ITM grant by the Hungarian National Research, Development and Innovation Office. JR is thankful to Semmelweis University for the EFOP-3.6.3-VEKOP-16-2017-00009 grant and to the Ministry of Innovation and Technology of Hungary from the National Research, Development and Innovation Fund for the ÚNKP-21-3-II-SE-1. Project no. 134196 has been implemented with the support provided by the Ministry of Innovation and Technology of Hungary from the National Research, Development and Innovation Fund, financed under the PD_20 funding scheme.

## Conflict of Interest

The authors declare that the research was conducted in the absence of any commercial or financial relationships that could be construed as a potential conflict of interest.

## Publisher's Note

All claims expressed in this article are solely those of the authors and do not necessarily represent those of their affiliated organizations, or those of the publisher, the editors and the reviewers. Any product that may be evaluated in this article, or claim that may be made by its manufacturer, is not guaranteed or endorsed by the publisher.

## Footnotes

^1^data source: https://spikeforest.flatironinstitute.org/.

^2^source: www.github.com/cortex-lab/phy.

^3^source: www.github.com/tridesclous/tridesclous.

^4^source: www.github.com/spyke/spyke.

## References

[B1] Abbott J. Ye T. Krenek K. Gertner R. S. Wu W. Jung H. S.. (2020). Extracellular recording of direct synaptic signals with a CMOS-nanoelectrode array. Lab. Chip 20, 3239–3248. 10.1039/D0LC00553C 32756639

[B2] Ahmadi N. Constandinou T. G. Bouganis C.-S. (2020). Improved spike-based brain-machine interface using bayesian adaptive kernel smoother and deep learning. techRxiv. 10, 29341–29356. 10.36227/techrxiv.12383600.v1

[B3] Ahmadpour S. Behrad A. Vega I. F. (2019). Dark neurons: a protective mechanism or a mode of death. J. Med. Histol. 3, 125–131. 10.21608/jmh.2020.40221.1081

[B4] Allen B. D. Moore-Kochlacs C. Bernstein J. G. Kinney J. P. Scholvin J. Seoane L. F.. (2018). Automated *in vivo* patch clamp evaluation of extracellular multielectrode array spike recording capability. J. Mater. Process. Technol. 1, 1–8. 10.1016/j.powtec.2016.12.055 29995597 PMC6295521

[B5] Aqrawe Z. Patel N. Vyas Y. Bansal M. Montgomery J. Travas-Sejdic J.. (2020). A simultaneous optical and electrical *in-vitro* neuronal recording system to evaluate microelectrode performance. PLoS ONE 15, e0237709. 10.1371/journal.pone.0237709 32817653 PMC7440637

[B6] Baldazzi G. Solinas G. Valle J. Del Barbaro M. Micera S. Raffo L.. (2020). Systematic analysis of wavelet denoising methods for neural signal processing. J. Neural Eng. 17, 066016. 10.1088/1741-2552/abc741 33142283

[B7] Barabino G. Baldazzi G. Sulas E. Carboni C. Raffo L. Pani D. (2017). Comparative evaluation of different wavelet thresholding methods for neural signal processing, in 2017 39th Annual International Conference of the IEEE Engineering in Medicine and Biology Society (Jeju: IEEE), 1042–1045. 10.1109/EMBC.2017.8037005 29060052

[B8] Barnett A. H. Magland J. F. Greengard L. F. (2016). Validation of neural spike sorting algorithms without ground-truth information. J. Neurosci. Methods 264, 65–77. 10.1016/j.jneumeth.2016.02.022 26930629

[B9] Berjis Z. K. Al-sulaifanie A. (2020). Neural spike sorting and classification. J. Duhok Univ. 23, 166–178. 10.26682/sjuod.2020.23.2.18

[B10] Bernert M. Yvert B. (2019). An attention-based spiking neural network for unsupervised spike-sorting. Int. J. Neural Syst. 29, 1–19. 10.1142/S0129065718500594 30776985

[B11] Bigelow J. Malone B. J. (2021). Extracellular voltage thresholds for maximizing information extraction in primate auditory cortex: implications for a brain computer interface. J. Neural Eng. 18, 036010. 10.1088/1741-2552/ab7c19 32126540 PMC12371764

[B12] Bjånes D. Fisher L. Gaunt R. Weber D. (2020). Heuristic Spike Sorting Tuner (HSST), a framework to determine optimal parameter selection for a generic spike sorting algorithm. bioRxiv. 10.1101/2020.05.21.108902

[B13] Boran E. Fedele T. Steiner A. Hilfiker P. Stieglitz L. Grunwald T.. (2020). Dataset of human medial temporal lobe neurons, scalp and intracranial EEG during a verbal working memory task. Sci. Data 7, 1–7. 10.1038/s41597-020-0364-3 31964868 PMC6972902

[B14] Brochier T. Zehl L. Hao Y. Duret M. Sprenger J. Denker M.. (2018). Data descriptor: massively parallel recordings in macaque motor cortex during an instructed delayed reach-to-grasp task. Sci. Data 5, 1–23. 10.1038/sdata.2018.55 29633986 PMC5892370

[B15] Buccino A. P. Einevoll G. T. (2021). MEArec: a fast and customizable testbench simulator for ground-truth extracellular spiking activity. Neuroinformatics 19, 185–204. 10.1007/s12021-020-09467-7 32648042 PMC7782412

[B16] Buccino A. P. Hagen E. Einevoll G. T. Hafliger P. D. Cauwenbergh G. (2018). Independent component analysis for fully automated multi-electrode array spike sorting, in 2018 40th Annual International Conference of the IEEE Engineering in Medicine and Biology Society (Honolulu, HI: IEEE), 2627–2630. 10.1109/EMBC.2018.8512788 30440947

[B17] Buccino A. P. Hurwitz C. L. Garcia S. Magland J. Siegle J. H. Hurwitz R.. (2020). Spikeinterface, a unified framework for spike sorting. Elife 9, 1–24. 10.7554/eLife.61834 33170122 PMC7704107

[B18] Buccino A. P. Kuchta M. Jæger K. H. Vefferstad N. T. Berthet P. Mardal K.-A.. (2019). How does the presence of neural probes affect extracellular potentials? J. Neural Eng. 16.2, 026030. 10.1088/1741-2552/ab03a1 30703758

[B19] Camuñas-Mesa L. A. Quiroga R. Q. (2013). A detailed and fast model of extracellular recordings. Neural Comput. 25, 1191–1212. 10.1162/NECO_a_00433 23470125

[B20] Carlson D. Carin L. (2019). Continuing progress of spike sorting in the era of big data. Curr. Opin. Neurobiol. 55, 90–96. 10.1016/j.conb.2019.02.007 30856552 PMC7702194

[B21] Caro-Martín C. R. Delgado-García J. M. Gruart A. Sánchez-Campusano R. (2018). Spike sorting based on shape, phase, and distribution features, and K-TOPS clustering with validity and error indices. Sci. Rep. 8, 33–38. 10.1038/s41598-018-35491-4 30542106 PMC6290782

[B22] Cetinkaya E. Gok S. Sahin M. (2018). Carbon fiber electrodes for *in vivo* spinal cord recordings, in 2018 40th Annual International Conference of the IEEE Engineering in Medicine and Biology Society (EMBC) (Honolulu, HI: IEEE), 5069–5072. 10.1109/EMBC.2018.8513408 30441480

[B23] Chah E. Hok V. Della-Chiesa A. Miller J. J. H. O'Mara S. M. Reilly R. B. (2011). Automated spike sorting algorithm based on Laplacian eigenmaps and k -means clustering. J. Neural Eng. 8, 016006. 10.1088/1741-2560/8/1/016006 21248378

[B24] Chaure F. J. Rey H. G. Quian Quiroga R. (2018). A novel and fully automatic spike-sorting implementation with variable number of features. J. Neurophysiol. 120, 1859–1871. 10.1152/jn.00339.2018 29995603 PMC6230803

[B25] Chen K. Jiang Y. Wu Z. Zheng N. Wang H. Hong H. (2021). HTsort: enabling fast and accurate spike sorting on multi-electrode arrays. Front. Comput. Neurosci. 15, 1–13. 10.3389/fncom.2021.657151 34234663 PMC8255361

[B26] Chiarion G. Mesin L. (2021). Resolution of spike overlapping by biogeography-based optimization. Electron. 10, 1469. 10.3390/electronics10121469

[B27] Choi J. R. Kim S. M. Ryu R. H. Kim S. P. Sohn J. W. (2018). Implantable neural probes for brain-machine interfaces - current developments and future prospects. Exp. Neurobiol. 27, 453–471. 10.5607/en.2018.27.6.453 30636899 PMC6318554

[B28] Chou M. C. H. (2021). On a simple and fast thresholding method for spike sorting, in AUTOMED -Automation in Medical Engineering 2021. 10.1088/0954-898X29932426

[B29] Chrobok L. Wojcik M. Klich J. D. Pradel K. Lewandowski M. H. Piggins H. D. (2021). Phasic neuronal firing in the rodent nucleus of the solitary tract *ex vivo*. Front. Physiol. 12, 638695. 10.3389/fphys.2021.638695 33762969 PMC7982836

[B30] Chung J. E. Joo H. R. Fan J. L. Liu D. F. Barnett A. H. Chen S.. (2020). High-density, long-lasting, and multi-region electrophysiological recordings using polymer electrode arrays. Neuron 101, 21–31. 10.1016/j.neuron.2018.11.002 30502044 PMC6326834

[B31] Chung J. E. Magland J. F. Barnett A. H. Tolosa V. M. Tooker A. C. Lee K. Y.. (2017). A fully automated approach to spike sorting. Neuron 95, 1381–1394.e6. 10.1016/j.neuron.2017.08.030 28910621 PMC5743236

[B32] Ciecierski K. A. (2020). Neural spike sorting using unsupervised adversarial learning, in International Symposium on Methodologies for Intelligent Systems, 192–202.

[B33] Cleaver-Stigum I. (2021). Clustering hippocampal neuron action potentials using autoencoders and autoencoder-kalman filtering for noise reduction (Dissertion). Worcester Polytechnic Institute, Worester, MA, United States.

[B34] Dai J. Zhang P. Sun H. Qiao X. Zhao Y. Ma J.. (2019). Reliability of motor and sensory neural decoding by threshold crossings for intracortical brain-machine interface. J. Neural Eng. 16, 036011. 10.1088/1741-2552/ab0bfb 30822756

[B35] Dallal A. H. Chen Y. Weber D. Mao Z. H. (2016). Dictionary learning for sparse representation and classification of neural spikes, in 2016 38th Annual International Conference of the IEEE Engineering in Medicine and Biology Society (EMBC) (Orlando, FL: IEEE), 3486–3489. 10.1109/EMBC.2016.7591479 28269050

[B36] Davey C. E. Soto-Breceda A. Shafton A. McAllen R. M. Furness J. B. Grayden D. B.. (2020). A new algorithm for drift compensation in multi-unit recordings of action potentials in peripheral autonomic nerves over time. J. Neurosci. Methods 338, 108683. 10.1016/j.jneumeth.2020.108683 32201350

[B37] De Dorigo D. Moranz C. Graf H. Marx M. Wendler D. Shui B.. (2018). Fully immersible subcortical neural probes with modular architecture and a delta-sigma ADC integrated under each electrode for parallel readout of 144 recording sites. IEEE J. Solid-State Circuits 53, 3111–3125. 10.1109/JSSC.2018.2873180

[B38] Dehnen G. Kehl M. S. Darcher A. Müller T. T. Macke J. H. Borger V.. (2021). Duplicate detection of spike events: a relevant problem in human single-unit recordings. Brain Sci. 11, 761. 10.3390/brainsci11060761 34201115 PMC8228483

[B39] Denker M. Einevoll G. T. Franke F. Grün S. Hagen E. Kerr J. N. D.. (2012). Validation of Analysis Methods, Workshop Report (Stockholm), 1–24.

[B40] Despouy E. Curot J. Reddy L. Nowak L. G. Deudon M. Sol J. C.. (2020). Recording local field potential and neuronal activity with tetrodes in epileptic patients. J. Neurosci. Methods 341, 108759. 10.1016/j.jneumeth.2020.108759 32389603

[B41] Diggelmann R. Fiscella M. Hierlemann A. Franke F. (2018). Automatic spike sorting for high-density microelectrode arrays. J. Neurophysiol. 120, 3155–3171. 10.1152/jn.00803.2017 30207864 PMC6314465

[B42] Dimitriadis G. Neto J. Aarts A. Alexandru A. Ballini M. Battaglia F.. (2018a). Why not record from every electrode with a CMOS scanning probe? bioRxiv 30, 275818. 10.1101/275818

[B43] Dimitriadis G. Neto J. P. Kampff A. R. (2018b). t-SNE visualization of large-scale neural recordings george. Neural Comput. 30, 1750–1774. 10.1162/neco_a_01097 29894653

[B44] Dragas J. Jäckel D. Hierlemann A. Franke F. (2017). Complexity optimization and high-throughput low-latency hardware implementation of a multi-electrode spike-sorting algorithm. IEEE Trans. Neural Syst. Rehabil. Eng. 23, 149–158. 10.1109/TNSRE.2014.2370510 25415989 PMC5421577

[B45] Endo D. Kobayashi R. Bartolo R. Averbeck B. B. Sugase-Miyamoto Y. Hayashi K.. (2021). A convolutional neural network for estimating synaptic connectivity from spike trains. Sci. Rep. 11, 1–18. 10.1038/s41598-021-91244-w 34103546 PMC8187444

[B46] Eom J. Park I. Y. Kim S. Jang H. Park S. Huh Y.. (2021). Deep-learned spike representations and sorting via an ensemble of auto-encoders. Neural Netw. 134, 131–142. 10.1016/j.neunet.2020.11.009 33307279

[B47] Ereifej E. S. Smith C. S. Meade S. M. Chen K. Feng H. Capadona J. R. (2018). The neuroinflammatory response to nanopatterning parallel grooves into the surface structure of intracortical microelectrodes. Adv. Funct. Mater. 28, 1–13. 10.1002/adfm.201704420

[B48] Evangelou A. (2020). Electrophysiology and spike sorting on neuronal in vitro extracellular recordings with multi-modal CMOS multi-electrode array chip (Master Thesis). Aristotle University Of Thessaloniki, Thessaloniki, Greece. Available online at: http://ikee.lib.auth.gr/record/324151/files/AlexandrosEvangelou.pdf

[B49] Farashi S. (2018). Spike detection using a multiresolution entropy based method. Biomed. Tech. 63, 361–376. 10.1515/bmt-2016-0182 28640748

[B50] Febinger H. Y. Dorval A. D. Rolston J. D. (2018). A sordid affair: spike sorting and data reproducibility. Sci. Times 82, 19–20. 10.1093/neuros/nyx590 29462436

[B51] Fedele T. Boran E. Chirkov V. Hilfiker P. Grunwald T. Stieglitz L.. (2021). Dataset of spiking and LFP activity invasively recorded in the human amygdala during aversive dynamic stimuli. Sci. Data 8, 1–6. 10.1038/s41597-020-00790-x 33446665 PMC7809031

[B52] Fiani B. Reardon T. Ayres B. Cline D. Sitto S. R. (2021). An examination of prospective uses and future directions of neuralink: the brain-machine interface. Cureus 13. 10.7759/cureus.14192 33936901 PMC8083990

[B53] Fiáth R. Márton A. L. Mátyás F. Pinke D. Márton G. Tóth K.. (2019a). Slow insertion of silicon probes improves the quality of acute neuronal recordings. Sci. Rep. 9, 1–17. 10.1038/s41598-018-36816-z 30643182 PMC6331571

[B54] Fiáth R. Meszéna D. Somogyvári Z. Boda M. Barthó P. Ruther P.. (2021). Recording site placement on planar silicon-based probes affects signal quality in acute neuronal recordings. Sci. Rep. 11, 1–18. 10.1038/s41598-021-81127-5 33479289 PMC7819990

[B55] Fiáth R. Raducanu B. C. Musa S. Andrei A. Lopez C. M. Welkenhuysen M.. (2019b). Fine-scale mapping of cortical laminar activity during sleep slow oscillations using high-density linear silicon probes. J. Neurosci. Methods 316, 58–70. 10.1016/j.jneumeth.2018.08.020 30144495

[B56] Fournier J. Mueller C. M. Shein-Idelson M. Hemberger M. Laurent G. (2016). Consensus-based sorting of neuronal spike waveforms. PLoS ONE 11, e0160494. 10.1371/journal.pone.0160494 27536990 PMC4990262

[B57] Francoeur M. J. Tang T. Fakhraei L. Wu X. Hulyalkar S. Cramer J.. (2021). Chronic, multi-site recordings supported by two low-cost, stationary probe designs optimized to capture either single unit or local field potential activity in behaving rats. Front. Psychiatry 12, 678103. 10.3389/fpsyt.2021.678103 34421671 PMC8374626

[B58] Franke F. Quian Quiroga R. Hierlemann A. Obermayer K. (2015). Bayes optimal template matching for spike sorting - combining fisher discriminant analysis with optimal filtering. J. Comput. Neurosci. 38, 439–459. 10.1007/s10827-015-0547-7 25652689 PMC4420847

[B59] Fu T. M. Hong G. Viveros R. D. Zhou T. Lieber C. M. (2017). Highly scalable multichannel mesh electronics for stable chronic brain electrophysiology. Proc. Natl. Acad. Sci. U.S.A. 114, E10046–E10055. 10.1073/pnas.1717695114 29109247 PMC5703340

[B60] Gainutdinov A. (2021). Method for analyzing the inhibition of cellular signals in the spike train format, in Saratov Fall Meeting 2020: Computations and Data Analysis: From Molecular Processes to Brain Functions (Saratov), 28. 10.1117/12.2591330

[B61] Gao L. Li F. Fu J. (2018). Neuronal spike sorting based on matching wavelet, in 2018 5th IEEE International Conference on Cloud Computing and Intelligence Systems (Beijing: IEEE), 53–57. 10.1109/CCIS.2018.8691204

[B62] Garcia S. Buccino A. P. Yger P. (2021). How do spike collisions affect spike sorting performance? bioRxiv. 10.1101/2021.11.29.470450 PMC9532018 36171060

[B63] Ghanbari A. Malyshev A. Volgushev M. Stevenson I. H. (2017). Estimating short-term synaptic plasticity from pre- and postsynaptic spiking. PLoS Comput. Biol. 13, 1–28. 10.1371/journal.pcbi.1005738 28873406 PMC5600391

[B64] Gherardi K. Toreyin H. (2021). A cortical extracellular simulation model to create synthetic neural recordings in A Cortical Extracellular Simulation Model to Create Synthetic Neural Recording, 465–468. 10.1109/NER49283.2021.9441104

[B65] Gong W. Sencar J. Bakkum D. J. Jäckel D. Obien M. E. J. Radivojevic M.. (2016). Multiple single-unit long-term tracking on organotypic hippocampal slices using high-density microelectrode arrays. Front. Neurosci. 10, 537. 10.3389/fnins.2016.00537 27920665 PMC5118563

[B66] Goshi N. Castagnola E. Vomeroc M. Guelic C. Cea C. Zucchini E.. (2018). Glassy carbon MEMS for novel origami-styled 3D integrated intracortical and epicortical neural probes. J. Micromech. Microeng. 27, 31. 10.1088/1361-6439/aab061

[B67] Govindan S. Batti L. Osterop S. F. Stoppini L. Roux A. (2021). Mass generation, neuron labeling, and 3D imaging of minibrains. Front. Bioeng. Biotechnol. 8, 582650. 10.3389/fbioe.2020.582650 33598450 PMC7883898

[B68] Guan S. Wang J. Gu X. Zhao Y. Hou R. Fan H.. (2019). Elastocapillary self-assembled neurotassels for stable neural activity recordings. Sci. Adv. 5, eaav2842. 10.1126/sciadv.aav2842 30944856 PMC6436924

[B69] Guido R. C. (2021). Nearly symmetric orthogonal wavelets for time-frequency-shape joint analysis: introducing the discrete shapelet transform's third generation (DST-III) for nonlinear signal analysis. Commun. Nonlinear Sci. Numer. Simul. 97, 1–12. 10.1016/j.cnsns.2020.105685

[B70] Güngör C. B. Mercier P. P. Töreyin H. (2021). Investigating well potential parameters on neural spike enhancement in a stochastic-resonance pre-emphasis algorithm. J. Neural Eng. 18, 046062. 10.1088/1741-2552/abfd0f 33915529

[B71] Güngör C. B. Töreyin H. (2020). Facilitating stochastic resonance as a pre-emphasis method for neural spike detection. J. Neural Eng. 17, 046047. 10.1088/1741-2552/abae8a 32945270

[B72] Guo X. Yu H. Kodama N. X. Wang J. Galán R. F. (2020). Fluctuation scaling of neuronal firing and bursting in spontaneously active brain circuits. Int. J. Neural Syst. 30, 1–16. 10.1142/S0129065719500175 31390911

[B73] Gupta I. Serb A. Khiat A. Trapatseli M. Prodromakis T. (2019). Spike sorting using non-volatile metal-oxide memristors. Faraday Discuss. 213, 511–520. 10.1039/C8FD00130H 30564810

[B74] Gupta I. Serb A. Khiat A. Zeitler R. Vassanelli S. Prodromakis T. (2016). Real-time encoding and compression of neuronal spikes by metal-oxide memristors. Nat. Commun. 7, 1–9. 10.1038/ncomms12805 27666698 PMC5052668

[B75] Guzman E. Cheng Z. Hansma P. K. Tovar K. R. Petzold L. R. Kosik K. S. (2021). Extracellular detection of neuronal coupling. Sci. Rep. 11, 1–11. 10.1038/s41598-021-94282-6 34282275 PMC8289866

[B76] Hadianpour M. Rezayat E. Dehaqani M. -R. (2021). High-performance computing framework based on distributed systems for large-scale neurophysiological data. Res. Squ. 1–14. 10.21203/rs.3.rs-136986/v119837177

[B77] Haessig G. Lesta D. G. Lenz G. Benosman R. Dudek P. (2020). A mixed-signal spatio-temporal signal classifier for on-sensor spike sorting, in 2020 IEEE International Symposium on Circuits and Systems (Seville: IEEE), 1–5. 10.1109/ISCAS45731.2020.9180442

[B78] Hagen E. Ness T. V. Khosrowshahi A. Sørensen C. Fyhn M. Hafting T.. (2015). ViSAPy: a Python tool for biophysics-based generation of virtual spiking activity for evaluation of spike-sorting algorithms. J. Neurosci. Methods 245, 182–204. 10.1016/j.jneumeth.2015.01.029 25662445

[B79] Hammad S. H. Kamavuako E. N. Farina D. Jensen W. (2016). Simulation of a real-time brain computer interface for detecting a self-paced hitting task. Neuromodulation 19, 804–811. 10.1111/ner.12478 27513737

[B80] Hara S. A. Kim B. J. Kuo J. T. W. Lee C. D. Meng E. Pikov V. (2016). Long-term stability of intracortical recordings using perforated and arrayed Parylene sheath electrodes. J. Neural Eng. 13, 066020. 10.1088/1741-2560/13/6/066020 27819256

[B81] Harris K. D. Quiroga R. Q. Freeman J. Smith S. L. (2016). Improving data quality in neuronal population recordings. Nat. Neurosci. 19, 1165–1174. 10.1038/nn.4365 27571195 PMC5244825

[B82] Hassan M. U. Veerabhadrappa R. Bhatti A. (2021). Efficient neural spike sorting using data subdivision and unification. PLoS ONE 16, e0245589. 10.1371/journal.pone.0245589 33566859 PMC7875432

[B83] Hassan M. U. Veerabhadrappa R. Zhang J. Bhatti A. (2020). Robust optimal parameter estimation (OPE) for unsupervised clustering of spikes using neural networks, in Robust Optimal Parameter Estimation (OPE) for Unsupervised Clustering of Spikes Using Neural Networks (Toronto, ON), 1286–1291. 10.1109/SMC42975.2020.9283347

[B84] He F. Lycke R. Ganji M. Xie C. Luan L. (2020). Ultraflexible neural electrodes for long-lasting intracortical recording. iScience 23, 101387. 10.1016/j.isci.2020.101387 32745989 PMC7398974

[B85] Hennig M. H. Hurwitz C. Sorbaro M. (2019). Scaling spike detection and sorting for next-generation electrophysiology. Adv. Neurobiol. 22, 171–184. 10.1007/978-3-030-11135-9_7 31073936

[B86] Hess-Dunning A. Tyler D. J. (2018). A mechanically-adaptive polymer nanocomposite-based intracortical probe and package for chronic neural recording. Micromachines 9, 583. 10.3390/mi9110583 30413034 PMC6265703

[B87] Hildebrandt K. J. Sahani M. Linden J. F. (2017). The impact of anesthetic state on spike-sorting success in the cortex: a comparison of ketamine and urethane anesthesia. Front. Neural Circuits 11, 95. 10.3389/fncir.2017.00095 29238293 PMC5712555

[B88] Hilgen G. Sorbaro M. Pirmoradian S. Muthmann J. O. Kepiro I. E. Ullo S.. (2017). Unsupervised spike sorting for large-scale, high-density multielectrode arrays. Cell Rep. 18, 2521–2532. 10.1016/j.celrep.2017.02.038 28273464

[B89] Hojjatinia S. Shoorehdeli M. A. Fatahi Z. Hojjatinia Z. Haghparast A. (2020). Improving the Izhikevich model based on rat basolateral amygdala and hippocampus neurons, and recognizing their possible firing patterns. Basic Clin. Neurosci. 11, 79–90. 10.32598/bcn.9.10.435 32483478 PMC7253815

[B90] Hong G. Lieber C. M. (2019). Novel electrode technologies for neural recordings. Nat. Rev. Neurosci. 20, 330–345. 10.1038/s41583-019-0140-6 30833706 PMC6531316

[B91] Horváth C. Tóth L. F. Ulbert I. Richárd F. (2021). Dataset of cortical activity recorded with high spatial resolution from anesthetized rats. Sci. Data 8, 1–14. 10.1038/s41597-021-00970-3 34267214 PMC8282648

[B92] Hu S. Zhang Q. Wang J. Chen Z. (2018). Real-time particle filtering and smoothing algorithms for detecting abrupt changes in neural ensemble spike activity. J. Neurophysiol. 119, 1394–1410. 10.1152/jn.00684.2017 29357468 PMC5966736

[B93] Huang L. Gan L. Ling B. W. K. (2021). A unified optimization model of feature extraction and clustering for spike sorting. IEEE Trans. Neural Syst. Rehabil. Eng. 29, 750–759. 10.1109/TNSRE.2021.3074162 33877983

[B94] Huang L. Ling B. W. Zeng Y. Gan L. (2020). Spike sorting based on low-rank and sparse representation, in 2020 IEEE International Conference on Multimedia and Expo (London: IEEE), 1–6. 10.1109/ICME46284.2020.9102837

[B95] Huang L. Ling B. W. K. Cai R. Zeng Y. He J. Chen Y. (2019). WMsorting: wavelet packets' decomposition and mutual information-based spike sorting method. IEEE Trans. Nanobiosci. 18, 283–295. 10.1109/TNB.2019.2909010 30951475

[B96] Huang Z. H. Wang Z. G. Lü X. Y. Li W. Y. Zhou Y. X. Shen X. Y.. (2016). The principle of the micro-electronic neural bridge and a prototype system design. IEEE Trans. Neural Syst. Rehabil. Eng. 24, 180–191. 10.1109/TNSRE.2015.2466659 26276996

[B97] Hunt D. L. Lai C. Smith R. D. Lee A. K. Harris T. D. Barbic M. (2019). Multimodal *in vivo* brain electrophysiology with integrated glass microelectrodes. Nat. Biomed. Eng. 3, 741–753. 10.1038/s41551-019-0373-8 30936430

[B98] Hurwitz C. Kudryashova N. Onken A. Hennig M. H. (2021). Building population models for large-scale neural recordings: opportunities and pitfalls. Curr. Opin. Neurobiol. 70, 64–73. Available online at: http://arxiv.org/abs/2102.01807 34411907 10.1016/j.conb.2021.07.003

[B99] Iniguez-Lomeli F. J. Bornat Y. Renaud S. Barron-Zambrano J. H. Rostro-Gonzalez H. (2021). A real-time FPGA-based implementation for detection and sorting of bio-signals. Neural Comput. Appl. 10.1007/s00521-021-05853-7

[B100] Irwin Z. T. Thompson D. E. Schroeder K. E. Tat D. M. Hassani A. Bullard A. J.. (2016). Enabling low-power, multi-modal neural interfaces through a common, low-bandwidth feature space. IEEE Trans. Neural Syst. Rehabil. Eng. 24, 521–531. 10.1109/TNSRE.2015.2501752 26600160

[B101] Isbister J. B. Reyes-Puerta V. Sun J. J. Horenko I. Luhmann H. J. (2021). Clustering and control for adaptation uncovers time-warped spike time patterns in cortical networks *in vivo*. Sci. Rep. 11, 1–20. 10.1038/s41598-021-94002-0 34326363 PMC8322153

[B102] Issar D. Williamson R. C. Khanna S. B. Smith M. A. (2020). A neural network for online spike classification that improves decoding accuracy. J. Neurophysiol. 123, 1472–1485. 10.1152/jn.00641.2019 32101491 PMC7191521

[B103] Jurczynski P. Le Cam S. Rossion B. Ranta R. (2021). Separating local and propagated contributors to the behnke-fried microelectrode recordings. BIOSIGNALS 2021 4, 343–350. 10.5220/0010349303430350

[B104] Kaku H. Ozturk M. Viswanathan A. Jimenez-Shahed J. Sheth S. Ince N. F. (2019). Grouping neuronal spiking patterns in the subthalamic nucleus of Parkinsonian patients, in 2019 41st Annual International Conference of the IEEE Engineering in Medicine and Biology Society (EMBC) (Berlin: IEEE), 4221–4224. 10.1109/EMBC.2019.8857418 31946800

[B105] Kalmykov A. Reddy J. W. Bedoyan E. Wang Y. Garg R. Rastogi S. K.. (2021). Bioelectrical interfaces with cortical spheroids in three-dimensions. J. Neural Eng. 18, 1–46. 10.1088/1741-2552/abf290 33770775

[B106] Kapucu F. E. Mäkinen M. E. L. Tanskanen J. M. A. Ylä-Outinen L. Narkilahti S. Hyttinen J. A. K. (2016). Joint analysis of extracellular spike waveforms and neuronal network bursts. J. Neurosci. Methods 259, 143–155. 10.1016/j.jneumeth.2015.11.022 26675487

[B107] Keshtkaran M. R. Yang Z. (2017). Noise-robust unsupervised spike sorting based on discriminative subspace learning with outlier handling. J. Neural Eng. 18, 14. 10.1088/1741-2552/aa6089 28198354

[B108] Kim C. Jeong J. Kim S. J. (2019). Recent progress on non-conventional microfabricated probes for the chronic recording of cortical neural activity. Sensors 19, 1–23. 10.3390/s19051069 30832357 PMC6427797

[B109] Kim G. H. Kim K. Lee E. An T. Choi W. S. Lim G.. (2018). Recent progress on microelectrodes in neural interfaces. Materials 11. 10.3390/ma11101995 30332782 PMC6213370

[B110] Klempír O. Krupička R. Krušek J. Dittert I. Petráková V. Petrák V.. (2020). Application of spike sorting algorithm to neuronal signals originated from boron doped diamond micro-electrode arrays. Physiol. Res. 69, 529–536. 10.33549/physiolres.934366 32469239 PMC8648311

[B111] Knieling S. Niediek J. Kutter E. Bostroem J. Elger C. E. Mormann F. (2017). An online adaptive screening procedure for selective neuronal responses. J. Neurosci. Methods 291, 36–42. 10.1016/j.jneumeth.2017.08.002 28826654

[B112] Knieling S. Sridharan K. S. Belardinelli P. Naros G. Weiss D. Mormann F.. (2016). An unsupervised online spike-sorting framework. Int. J. Neural Syst. 26. 10.1142/S0129065715500422 26711713

[B113] Kodandaramaiah S. B. Holst G. L. Wickersham I. R. Singer A. C. Franzesi G. T. McKinnon M. L.. (2016). Assembly and operation of the autopatcher for automated intracellular neural recording *in vivo*. Nat. Protoc. 11, 634–654. 10.1038/nprot.2016.007 26938115 PMC4877510

[B114] Kozák G. Földi T. Berényi A. (2020). Spike-and-wave discharges are not pathological sleep spindles, network-level aspects of age-dependent absence seizure development in rats. eNeuro 7. 10.1523/ENEURO.0253-19.2019 31862790 PMC6944477

[B115] Kuperstein M. (2021). Relating electrode impedance and recording quality in the rat hippocampus. Honor. Sch. Theses. 76.

[B116] Laboy-Juárez K. J. Ahn S. Feldman D. E. (2019). A normalized template matching method for improving spike detection in extracellular voltage recordings. Sci. Rep. 9, 1–12. 10.1038/s41598-019-48456-y 31427615 PMC6700190

[B117] Larionov P. Juergens T. Schanze T. (2019). Correlation-based spike sorting of multivariate data. Curr. Dir. Biomed. Eng. 5, 113–116. 10.1515/cdbme-2019-0029

[B118] Lee J. H. Carlson D. Shokri H. Yao W. Goetz G. Hagen E.. (2017). YASS: Yet another spike sorter, in 31st Conference on Neural Information Processing Systems (Long Beach, CA), 4003–4013. 10.1101/151928

[B119] Lee K. H. Ni Y. L. Colonell J. Karsh B. Putzeys J. Pachitariu M.. (2021). Electrode pooling can boost the yield of extracellular recordings with switchable silicon probes. Nat. Commun. 12, 1–14. 10.1038/s41467-021-25443-4 34475396 PMC8413349

[B120] Lefebvre B. Ygera P. Marrea O. (2016). Recent progress in multi-electrode spike sorting methods. J. Physiol. Paris 110, 327–335. 10.1016/j.jphysparis.2017.02.005 28263793 PMC5581741

[B121] Leibig C. Wachtler T. Zeck G. (2016). Unsupervised neural spike sorting for high-density microelectrode arrays with convolutive independent component analysis. J. Neurosci. Methods 271, 1–13. 10.1016/j.jneumeth.2016.06.006 27317497

[B122] Li H. G. Song R. Q. Liu J. W. (2018). Low-dimensional feature fusion strategy for overlapping neuron spike sorting. Neurocomputing 281, 152–159. 10.1016/j.neucom.2017.12.004

[B123] Li J. Chen X. Li Z. (2019). Spike detection and spike sorting with a hidden Markov model improves offline decoding of motor cortical recordings. J. Neural Eng. 16. 10.1088/1741-2552/aaeaae 30523823

[B124] Li J. Li Z. (2017). Sums of spike waveform features for motor decoding. Front. Neurosci. 11, 406. 10.3389/fnins.2017.00406 28769745 PMC5513987

[B125] Li M. Liang Y. Yang L. Wang H. Yang Z. Zhao K.. (2020a). Automatic bad channel detection in implantable brain-computer interfaces using multimodal features based on local field potentials and spike signals. Comput. Biol. Med. 116, 103572. 10.1016/j.compbiomed.2019.103572 32001011

[B126] Li W. Qin S. Lu Y. Wang H. Xu Z. Wu T. (2021). A facile and comprehensive algorithm for electrical response identification in mouse retinal ganglion cells. PLoS ONE 16, e0246547. 10.1371/journal.pone.0246547 33705406 PMC7951861

[B127] Li Z. Wang Y. Zhang N. Li X. (2020b). An accurate and robust method for spike sorting based on convolutional neural networks. Brain Sci. 10, 1–16. 10.3390/brainsci10110835 33187098 PMC7696441

[B128] Lieb F. Stark H. G. Thielemann C. (2017). A stationary wavelet transform and a time-frequency based spike detection algorithm for extracellular recorded data. J. Neural Eng. 14. 10.1088/1741-2552/aa654b 28272020

[B129] Liu D. Lengyel M. (2021). A universal probabilistic spike count model reveals ongoing modulation of neural variability. bioRxiv. 34. 10.1101/2021.06.27.450063

[B130] Liu Y. Luan S. Williams I. Rapeaux A. Constandinou T. G. (2017). A 64-channel versatile neural recording soc with activity-dependent data throughput. IEEE Trans. Biomed. Circuits Syst. 11, 1344–1355. 10.1109/TBCAS.2017.2759339 29293425

[B131] Liu Y. Pereira J. L. Constandinou T. G. (2018). Event-driven processing for hardware-efficient neural spike sorting. J. Neural Eng. 15. 10.1088/1741-2552/aa9124 28978779

[B132] Lu L. Popeney B. Dickman J. D. Angelaki D. E. (2018). Construction of an improved multi-tetrode hyperdrive for large-scale neural recording in behaving rats. J. Vis. Exp. 135, 1–10. 10.3791/57388 29806835 PMC6101149

[B133] Luan S. Williams I. Maslik M. Liu Y. Felipe De C. Jackson A.. (2018). Compact standalone platform for neural recording with real-time spike sorting and data logging. J. Neural Eng. 15. 10.1088/1741-2552/aabc23 29623905

[B134] Magland J. Jun J. J. Lovero E. Morley A. J. Hurwitz C. L. Buccino A. P.. (2020). Spikeforest, reproducible web-facing ground-truth validation of automated neural spike sorters. Elife 9, 1–22. 10.7554/eLife.55167 32427564 PMC7237210

[B135] Mahallati S. Bezdek J. C. Popovic M. R. Valiante T. A. (2019). Cluster tendency assessment in neuronal spike data. PLoS ONE 14, e0224547. 10.1371/journal.pone.0224547 31714913 PMC6850537

[B136] Mahmud M. Vassanelli S. (2019). Open-source tools for processing and analysis of *in vitro* extracellular neuronal signals. Adv. Neurobiol. 22, 233–250. 10.1007/978-3-030-11135-9_10 31073939

[B137] Malik M. H. Saeed M. Kamboh A. M. (2016). Automatic threshold optimization in nonlinear energy operator based spike detection, in 2016 38th Annual International Conference of the IEEE Engineering in Medicine and Biology Society (EMBC) (Orlando, FL: IEEE), 774–777. 10.1109/EMBC.2016.7590816 28268441

[B138] Markanday A. Bellet J. Bellet M. E. Inoue J. Hafed Z. M. Thier P. (2020). Using deep neural networks to detect complex spikes of cerebellar Purkinje cells. J. Neurophysiol. 123, 2217–2234. 10.1152/jn.00754.2019 32374226

[B139] Marques-Smith A. Neto J. P. Lopes G. Nogueira J. Calcaterra L. Frazao J.. (2018). Recording from the same neuron with high-density CMOS probes and patch-clamp: A ground-truth dataset and an experiment in collaboration. bioRxiv [Preprint]. 10.1101/370080

[B140] Matsumoto S. (2020). Enhanced responsiveness of cortical neurons during sleep (Ph. D. Dissertion). University of Tsukuba, Tsukuba, Japan.

[B141] Mena G. E. Grosberg L. E. Madugula S. Hottowy P. Litke A. Cunningham J.. (2017). Electrical stimulus artifact cancellation and neural spike detection on large multi-electrode arrays. PLoS Comput. Biol. 13, e1005842. 10.1371/journal.pcbi.1005842 29131818 PMC5703587

[B142] Metcalfe B. W. Clarke C. T. Donaldson N. Taylor J. (2017). A new method for neural spike alignment: the centroid filter. IEEE Trans. Neural Syst. Rehabil. Eng. 25, 1988–1997. 10.1109/TNSRE.2017.2716822 28641265

[B143] Metcalfe B. W. Hunter A. J. Graham-Harper-Cater J. E. Taylor J. T. (2021). Array processing of neural signals recorded from the peripheral nervous system for the classification of action potentials. J. Neurosci. Methods 347, 108967. 10.1016/j.jneumeth.2020.108967 33035576

[B144] Milo V. Malavena G. Compagnoni C. M. Ielmini D. (2020). Memristive and CMOS devices for neuromorphic computing. Materials 13, 166. 10.3390/ma13010166 31906325 PMC6981548

[B145] Mizuhiki T. Setogawa T. Shidara M. (2020). Reverse-filtering on extracellular action potential for waveform analysis. Neurosci. Res. 160, 1–10. 10.1016/j.neures.2019.10.009 31626824

[B146] Moghaddasi M. Aliyari Shoorehdeli M. Fatahi Z. Haghparast A. (2020). Unsupervised automatic online spike sorting using reward-based online clustering. Biomed. Signal Process. Control 56, 101701. 10.1016/j.bspc.2019.101701

[B147] Mokri Y. Salazar R. F. Goodell B. Baker J. Gray C. M. Yen S. C. (2017). Sorting overlapping spike waveforms from electrode and tetrode recordings. Front. Neuroinform. 11, 53. 10.3389/fninf.2017.00053 28860985 PMC5562672

[B148] Mondragón-González S. L. Burguière E. (2017). Bio-inspired benchmark generator for extracellular multi-unit recordings. Sci. Rep. 7, 1–13. 10.1038/srep43253 28233819 PMC5324125

[B149] Mukhopadhyay A. K. Chakrabarti I. Basu A. Sharad M. (2018). Power efficient Spiking Neural Network Classifier based on memristive crossbar network for spike sorting application. Arxiv. 10.48550/arXiv.1802.09047

[B150] Muratore D. G. Tandon P. Wootters M. Chichilnisky E. J. Mitra S. Murmann B. (2019). A data-compressive wired-or readout for massively parallel neural recording, in IEEE Transactions on Biomedical Circuits and Systems (Sapporo). 10.1109/ISCAS.2019.8702387 31425051

[B151] Musk E. (2019). An integrated brain-machine interface platform with thousands of channels. J. Med. Internet Res. 21, 11. 10.2196/16194 31642810 PMC6914248

[B152] Muthmann J. O. Amin H. Sernagor E. Maccione A. Panas D. Berdondini L.. (2015). Spike detection for large neural populations using high density multielectrode arrays. Front. Neuroinform. 9, 28. 10.3389/fninf.2015.00028 26733859 PMC4683190

[B153] Navratilova Z. Godfrey K. B. McNaughton B. L. (2016). Grids from bands, or bands from grids? An examination of the effects of single unit contamination on grid cell firing fields. J. Neurophysiol. 115, 992–1002. 10.1152/jn.00699.2015 26683071

[B154] Negri J. Menon V. Young-Pearse T. L. (2020). Assessment of spontaneous neuronal activity *In vitro* using multi-well multi-electrode arrays: implications for assay development. eNeuro 7, 1–27. 10.1523/ENEURO.0080-19.2019 31896559 PMC6984810

[B155] Neto J. P. Baião P. Lopes G. Frazão J. Nogueira J. Fortunato E.. (2018). Does impedance matter when recording spikes with polytrodes? Front. Neurosci. 12, 715. 10.3389/fnins.2018.00715 30349453 PMC6188074

[B156] Neto J. P. Lopes G. Frazão J. Nogueira J. Lacerda P. Baião P.. (2016). Validating silicon polytrodes with paired juxtacellular recordings: method and dataset. J. Neurophysiol. 116, 892–903. 10.1152/jn.00103.2016 27306671 PMC5002440

[B157] Neumann A. R. Raedt R. Steenland H. W. Sprengers M. Bzymek K. Navratilova Z.. (2017). Involvement of fast-spiking cells in ictal sequences during spontaneous seizures in rats with chronic temporal lobe epilepsy. Brain 140, 2355–2369. 10.1093/brain/awx179 29050390 PMC6248724

[B158] Noce E. Dellacasa Bellingegni A. Ciancio A. L. Sacchetti R. Davalli A. Guglielmelli E.. (2019). EMG and ENG-envelope pattern recognition for prosthetic hand control. J. Neurosci. Methods 311, 38–46. 10.1016/j.jneumeth.2018.10.004 30316891

[B159] Oghazian M. Khadem A. Esghaei M. (2020). Functional classification of neurons in mouse hippocampus based on spike waveforms in extracellular recordings, in 2020 28th Iranian Conference on Electrical Engineering (Tabriz). 10.1109/ICEE50131.2020.9260737

[B160] Oh S. Han S. Youn I. (2017). Real-time neural signal sensing and spike sorting system using a modified zero-crossing feature with highly efficient data computation and transmission. Sensors Mater. 29, 1031–1042. 10.18494/SAM.2017.1552

[B161] Okkesim S. Rastogi S. Christ O. Hubka P. Rosskothen-Kuhl N. Hofmann U. G. (2021). Diversify your workflow! - an inconvenient suggestion to analyze spike data from intracranial recordings. bioRxiv. 10.1101/2021.03.10.434718

[B162] Okun M. Lak A. Carandini M. Harris K. D. (2016). Long term recordings with immobile silicon probes in the mouse cortex. PLoS ONE 11, e0151180. 10.1371/journal.pone.0151180 26959638 PMC4784879

[B163] Pachitariu M. Steinmetz N. Kadir S. Carandini M. Kenneth D. H. (2016). Kilosort: realtime spike-sorting for extracellular electrophysiology with hundreds of channels, in Advances in Neural Information Processing Systems, 29. 10.1101/061481

[B164] Pagin M. (2021). Data compression of neural spike signals (Dissertion). Universität Ulm, Ulm, Germany.

[B165] Pagin M. Ortmanns M. (2017). Evaluation of logarithmic vs. linear ADCs for neural signal acquisition and reconstruction, in Evaluation of Logarithmic vs. Linear ADCs for Neural Signal Acquisition and Reconstruction (Jeju), 4387–4390. 10.1109/EMBC.2017.8037828 29060869

[B166] Pagin M. Ortmanns M. (2018). Study of compressed sensing and predictor techniques for the compression of neural signals under the influence of noise, in 2018 40th Annual International Conference of the IEEE Engineering in Medicine and Biology Society (EMBC) (Honolulu, HI: IEEE), 1102–1105. 10.1109/EMBC.2018.8512469 30440582

[B167] Pakman A. Wang Y. Mitelut C. Lee J. H. Paninski L. (2020). Neural clustering processes, in 37th International Conference on Machine Learning, ICML 2020 Part F16814 (Vienna), 7411–7421.

[B168] Park I. Y. Eom J. Jang H. Kim S. Park S. Huh Y.. (2020). Deep learning-based template matching spike classification for extracellular recordings. Appl. Sci. 10, 301. 10.3390/app10010301

[B169] Park J. Kim G. Jung S. D. (2017). A 128-channel FPGA-based real-time spike-sorting bidirectional closed-loop neural interface system. IEEE Trans. Neural Syst. Rehabil. Eng. 25, 2227–2238. 10.1109/TNSRE.2017.2697415 28459692

[B170] Pastor J. Vega-Zelaya L. (2020). Features of action potentials from identified thalamic nuclei in anesthetized patients. Brain Sci. 10, 1–19. 10.3390/brainsci10121002 33348660 PMC7766545

[B171] Pedreira C. Martinez J. Ison M. J. Quian Quiroga R. (2012). How many neurons can we see with current spike sorting algorithms? J. Neurosci. Methods 211, 58–65. 10.1016/j.jneumeth.2012.07.010 22841630 PMC3657693

[B172] Pérez-Ortega J. Araya J. Ibaceta C. Herzog R. Escobar M.-J. Peña-Ortega F.. (2021). Parallel processing of natural images by overlapping retinal neuronal ensembles. bioRxiv. 10.1101/2021.02.22.432289

[B173] Petersen P. C. Siegle J. H. Steinmetz N. A. Mahallati S. Buzsáki G. (2020). CellExplorer: a graphical user interface and a standardized pipeline for visualizing and characterizing single neurons. bioRxiv. 10.1101/2020.05.07.083436 PMC8602784 34592168

[B174] Petrantonakis P. C. Poirazi P. (2017). A novel and simple spike sorting implementation. IEEE Trans. Neural Syst. Rehabil. Eng. 25, 323–333. 10.1109/TNSRE.2016.2640858 28113325

[B175] Pimenta S. Rodrigues J. A. Machado F. Ribeiro J. F. Maciel M. J. Bondarchuk O.. (2021). Double-layer flexible neural probe with closely spaced electrodes for high-density *in vivo* brain recordings. Front. Neurosci. 15, 663174. 10.3389/fnins.2021.663174 34211364 PMC8239195

[B176] Pregowska A. Casti A. Kaplan E. Wajnryb E. Szczepanski J. (2019). Information processing in the LGN: a comparison of neural codes and cell types. Biol. Cybern. 113, 453–464. 10.1007/s00422-019-00801-0 31243531 PMC6658673

[B177] Quian R. Q. Nadasdy Z. (2004). Unsupervised spike detection and sorting with wavelets and superparamagnetic clustering. Neural Comput. 16, 1661–1687. 10.1162/089976604774201631 15228749

[B178] Rácz M. Liber C. Nemeth E. Fiath R. Rokai J. Harmathi I.. (2020). Spike detection and sorting with deep learning. J. Neural Eng. 17, 016038. 10.1088/1741-2552/ab4896 31561235

[B179] Radmanesh M. Rezaei A. A. Hashemi A. Jalili M. (2021). Online spike sorting via deep contractive autoencoder. bioRxiv. 10.1101/2021.04.23.441225 36041279

[B180] Raghavan M. Fee D. Barkhaus P. E. (2019). Generation and propagation of the action potential, in Handbook of Clinical Neurology, eds Levin K. H. Chauvel P. (Amsterdam: Elsevier B.V.), 3–22. 10.1016/B978-0-444-64032-1.00001-1 31277855

[B181] Rahiminejad E. Azad F. Parvizi-Fard A. Amiri M. Linares-Barranco B. (2021). A neuromorphic CMOS circuit with self-repairing capability. IEEE Trans. Neural Networks Learn. Syst. 33, 2246–2258. 10.1109/TNNLS.2020.3045019 33417568

[B182] Ravikumar S. (2021). Abstract A 3D-printed Fat-IBC-enabled prosthetic arm?: Control based on brain neuronal data (Dissertion). Uppsala University, Uppsala, Sweden.

[B183] Reber T. P. Bausch M. Mackay S. Boström J. Elger C. E. Mormann F. (2019). Representation of abstract semantic knowledge in populations of human single neurons in the medial temporal lobe. PLoS Biol. 17, e3000290. 10.1371/journal.pbio.3000290 31158216 PMC6564037

[B184] Regalia G. Coelli S. Biffi E. Ferrigno G. Pedrocchi A. (2016). A framework for the comparative assessment of neuronal spike sorting algorithms towards more accurate off-line and on-line microelectrode arrays data analysis. Comput. Intell. Neurosci. 2016. 10.1155/2016/8416237 27239191 PMC4863096

[B185] Rey H. G. Pedreira C. Quian Quiroga R. (2015). Past, present and future of spike sorting techniques. Brain Res. Bull. 119, 106–117. 10.1016/j.brainresbull.2015.04.007 25931392 PMC4674014

[B186] Rezaei M. R. Arai K. Frank L. M. Eden U. T. Yousefi A. (2021). Real-time point process filter for multidimensional decoding problems using mixture models. J. Neurosci. Methods 348, 109006. 10.1016/j.jneumeth.2020.109006 33232686 PMC8828672

[B187] Richner T. J. Brodnick S. K. Thongpang S. (2019). Phase relationship between micro- electrocorticography and cortical neurons. J. Neural Eng. 16. 10.1088/1741-2552/ab335b 31318702

[B188] Rivnay J. Wang H. Fenno L. Deisseroth K. Malliaras G. G. (2017). Next-generation probes, particles, and proteins for neural interfacing. Sci. Adv. 3, 1–20. 10.1126/sciadv.1601649 28630894 PMC5466371

[B189] Rodriguez-Collado A. Rueda C. (2021). A simple parametric representation of the Hodgkin-Huxley Model Author summary. PLoS One 16, e0254152. 10.1371/journal.pone.0254152 34292948 PMC8297874

[B190] Rokai J. Rácz M. Fiáth R. Ulbert I. Márton G. (2021). Elvisort: encoding latent variables for instant sorting, an artificial intelligence-based end-to-end solution. J. Neural Eng. 18. 10.1088/1741-2552/abf521 33823497

[B191] Rosenberg D. M. Horn C. C. (2016). Neurophysiological analytics for all! Free open-source software tools for documenting, analyzing, visualizing, and sharing using electronic notebooks. J. Neurophysiol. 116, 252–262. 10.1152/jn.00137.2016 27098025 PMC4969392

[B192] Rossant C. Kadir S. N. Goodman D. F. M. Schulman J. Hunter M. L. D. Saleem A. B.. (2016). Spike sorting for large, dense electrode arrays. Nat. Neurosci. 19, 634–641. 10.1038/nn.4268 26974951 PMC4817237

[B193] Rossi-Pool R. Romo R. (2019). Low dimensionality, high robustness in neural population dynamics. Neuron 103, 177–179. 10.1016/j.neuron.2019.06.021 31319044

[B194] Rutishauser U. Schuman E. M. Mamelak A. N. (2006). Online detection and sorting of extracellularly recorded action potentials in human medial temporal lobe recordings, *in vivo*. J. Neurosci. Methods 154, 204–224. 10.1016/j.jneumeth.2005.12.033 16488479

[B195] Sacher W. D. Chen F.-D. Moradi-Chameh H. Liu X. Almog I. F. Lordello T.. (2021). Optical phased array neural probes for beam-steering in brain tissue. Arxiv. 10.48550/arXiv.2108.04933 35230293

[B196] Saeed M. Khan A. A. Kamboh A. M. (2017). Comparison of classifier architectures for online neural spike sorting. IEEE Trans. Neural Syst. Rehabil. Eng. 25, 334–344. 10.1109/TNSRE.2016.2641499 28029625

[B197] Saggese G. Tambaro M. Vallicelli E. A. Strollo A. G. M. Vassanelli S. Baschirotto A.. (2021). Comparison of sneo-based neural spike detection algorithms for implantable multi-transistor array biosensors. Electron. 10, 1–17. 10.3390/electronics10040410

[B198] Sahasrabuddhe K. Khan A. A. Singh A. P. Stern T. M. Ng Y. Tadić A.. (2021). The Argo: a high channel count recording system for neural recording *in vivo*. J. Neural Eng. 18. 10.1088/1741-2552/abd0ce 33624614 PMC8607496

[B199] Saif-Ur-Rehman M. Ali O. Dyck S. Lienkämper R. Metzler M. Parpaley Y.. (2021). SpikeDeep-classifier: a deep-learning based fully automatic offline spike sorting algorithm. J. Neural Eng. 18. 10.1088/1741-2552/abc8d4 33166944

[B200] Saif-Ur-Rehman M. Lienk mper R. Parpaley Y. Wellmer J. Liu C. Lee B.. (2019). SpikeDeeptector: a deep-learning based method for detection of neural spiking activity. J. Neural Eng. 16. 10.1088/1741-2552/ab1e63 31042684

[B201] Salman H. Grover J. Shankar T. (2018). Hierarchical reinforcement learning for sequencing behaviors. Neural Comput. 2733, 2709–2733. 10.1162/neco_a_01113 30021083

[B202] Salmasi M. Büttner U. Glasauer S. (2016). Fractal dimension analysis for spike detection in low SNR extracellular signals. J. Neural Eng. 13. 10.1088/1741-2560/13/3/036004 27064604

[B203] Saunier V. Flahaut E. Blatché M. C. Bergaud C. Maziz A. (2020). Carbon nanofiber-PEDOT composite films as novel microelectrode for neural interfaces and biosensing. Biosens. Bioelectron. 165. 10.1016/j.bios.2020.112413 32729532

[B204] Schaffer L. Nagy Z. Kincses Z. Fiath R. Ulbert I. (2021). Spatial information based OSort for real-time spike sorting using FPGA, in 2017 *IEEE International Symposium on Circuits and Systems* (IEEE), 99–108. 10.1109/TBME.2020.2996281 32746008

[B205] Schaffer L. Nagy Z. Kineses Z. Fiath R. (2017). FPGA-based neural probe positioning to improve spike sorting with OSort algorithm. Proc. IEEE Int. Symp. Circuits Syst. 10.1109/ISCAS.2017.8050608

[B206] Schiavone P. D. Rossi D. Liu Y. Benatti S. Luan S. Williams I.. (2020). Neuro-PULP: a paradigm shift towards fully programmable platforms for neural interfaces, in 2020 2nd IEEE International Conference on Artificial Intelligence Circuits and Systems (Genoa: IEEE), 50–54. 10.1109/AICAS48895.2020.9073920

[B207] Scholvin J. Kinney J. P. Bernstein J. G. Moore-Kochlacs C. Kopell N. Fonstad C. G.. (2016). Close-packed silicon microelectrodes for scalable spatially oversampled neural recording. IEEE Trans. Biomed. Eng. 63, 120–130. 10.1109/TBME.2015.2406113 26699649 PMC4692190

[B208] Sedaghat-Nejad E. Amin Fakharian M. Pi J. Hage P. Kojima Y. Ohmae S.. (2021). P-sort: an open-source software for cerebellar neurophysiology. bioRxiv. 126, 1055–1075. 10.1101/2021.03.16.435644 34432996 PMC8560425

[B209] Shaeri M. A. Sodagar A. M. (2020). A framework for on-implant spike sorting based on salient feature selection. Nat. Commun. 11, 1–9. 10.1038/s41467-020-17031-9 32606311 PMC7327047

[B210] Shan K. Q. Lubenov E. V. Siapas A. G. (2017). Model-based spike sorting with a mixture of drifting t- distributions. J. Neurosci. Methods 288, 82–98. 10.1016/j.jneumeth.2017.06.017 28652008 PMC5563448

[B211] Shibue R. Komaki F. (2017). Firing rate estimation using infinite mixture models and its application to neural decoding. J. Neurophysiol. 118, 2902–2913. 10.1152/jn.00818.2016 28794199 PMC5686235

[B212] Shin S. Kim J. H. Jeong J. Gwon T. M. Lee S. H. Kim S. J. (2017). Novel four-sided neural probe fabricated by a thermal lamination process of polymer films. J. Neurosci. Methods 278, 25–35. 10.1016/j.jneumeth.2016.12.017 28040494

[B213] Shmoel N. Rabieh N. Ojovan S. M. Erez H. Maydan E. Spira M. E. (2016). Multisite electrophysiological recordings by self-assembled loose-patch-like junctions between cultured hippocampal neurons and mushroom-shaped microelectrodes. Sci. Rep. 6, 1–11. 10.1038/srep27110 27256971 PMC4891817

[B214] Smith L. S. Mtetwa N. (2007). A tool for synthesizing spike trains with realistic interference. J. Neurosci. Methods 159, 170–180. 10.1016/j.jneumeth.2006.06.019 16887194

[B215] Smith T. M. Lee D. Bradley K. McMahon S. B. (2020). Methodology for quantifying excitability of identified projection neurons in the dorsal horn of the spinal cord, specifically to study spinal cord stimulation paradigms. J. Neurosci. Methods 330, 108479. 10.1016/j.jneumeth.2019.108479 31705935

[B216] Soleymankhani A. Shalchyan V. (2021). A new spike sorting algorithm based on continuous wavelet transform and investigating its effect on improving neural decoding accuracy. Neuroscience 468, 139–148. 10.1016/j.neuroscience.2021.05.036 34102262

[B217] Sonia T. Sadtler P. Batista A. Chase S. Ventura V. (2014). To sort or not to sort: the impact of spike-sorting on neural decoding performance. J. Neural Eng. 11. 10.1088/1741-2560/11/5/056005 25082508 PMC4454741

[B218] Sousa A. R. dos S. Garcia N. L. Vidakovic B. (2021). Bayesian wavelet shrinkage with beta priors. Comput. Stat. 36, 1341–1363. 10.1007/s00180-020-01048-1

[B219] Souza B. C. Lopes-dos-Santos V. Bacelo J. Tort A. B. L. (2019). Spike sorting with Gaussian mixture models. Sci. Rep. 9, 1–14. 10.1038/s41598-019-39986-6 30842459 PMC6403234

[B220] Steinmetz N. A. Aydin C. Lebedeva A. Okun M. Pachitariu M. Bauza M.. (2021). Neuropixels 2.0: a miniaturized high-density probe for stable, long-term brain recordings. Science 372. 10.1126/science.abf4588 33859006 PMC8244810

[B221] Steinmetz P. N. (2017). Comparison of combined spike detection and clustering using mutual information. J. Neurosci. Methods 291, 166–175. 10.1016/j.jneumeth.2017.08.009 28827163

[B222] Steinmetz P. N. (2020). Estimates of distributed coding of visual objects by single neurons in the human brain depend on which spike sorting technique is used. J. Neural Eng. 17. 10.1088/1741-2552/ab6cb8 31951220

[B223] Sukiban J. Voges N. Dembek T. A. Pauli R. Visser-Vandewalle V. Denker M.. (2019). Evaluation of spike sorting algorithms: application to human subthalamic nucleus recordings and simulations. Neuroscience 414, 168–185. 10.1016/j.neuroscience.2019.07.005 31299347

[B224] Sun S. H. Almasi A. Yunzab M. Zehra S. Hicks D. G. Kameneva T.. (2021). Analysis of extracellular spike waveforms and associated receptive fields of neurons in cat primary visual cortex. J. Physiol. 599, 2211–2238. 10.1113/JP280844 33501669

[B225] Swindale N. V. Mitelut C. Murphy T. H. Spacek M. A. (2017). A visual guide to sorting electrophysiological recordings using 'SpikeSorter.' J. Vis. Exp. 2017, 1–15. 10.3791/55217 28287541 PMC5408889

[B226] Swindale N. V. Spacek M. A. (2014). Spike sorting for polytrodes: a divide and conquer approach. Front. Syst. Neurosci. 8, 6. 10.3389/fnsys.2014.00006 24574979 PMC3918743

[B227] Swindale N. V. Spacek M. A. (2016). Verification of multichannel electrode array integrity by use of cross-channel correlations. J. Neurosci. Methods 263, 95–102. 10.1016/j.jneumeth.2016.02.009 26875661

[B228] Szymanski L. J. Kellis S. Liu C. Y. Jones K. T. Andersen R. A. Commins D.. (2021). Neuropathological effects of chronically implanted, intracortical microelectrodes in a tetraplegic patient. J. Neural Eng. 18. 10.1088/1741-2552/ac127e 34314384

[B229] Tam W. K. Yang Z. (2018). Neural parallel engine: a toolbox for massively parallel neural signal processing. J. Neurosci. Methods 301, 18–33. 10.1016/j.jneumeth.2018.03.004 29530617

[B230] Tambaro M. Bisio M. Maschietto M. Leparulo A. Vassanelli S. (2021). FPGA design integration of a 32-microelectrodes low-latency spike detector in a commercial system for intracortical recordings. Digital 1, 34–53. 10.3390/digital1010003

[B231] Tambaro M. Vallicelli E. A. Saggese G. Strollo A. Baschirotto A. Vassanelli S. (2020). Evaluation of *in vivo* spike detection algorithms for implantable MTA brain-silicon interfaces. J. Low Power Electron. Appl. 10, 1–12. 10.3390/jlpea10030026

[B232] Tao L. Weber K. Arai K. Eden U. (2018). A common goodness-of-fit framework for neural population models using marked point process time-rescaling. J. Comput 45, 147–162. 10.1007/s10827-018-0698-4 30298220 PMC6208891

[B233] Tariq T. Satti M. H. Kamboh H. M. Saeed M. Kamboh A. M. (2019). Computationally efficient fully-automatic online neural spike detection and sorting in presence of multi-unit activity for implantable circuits. Comput. Methods Programs Biomed. 179, 104986. 10.1016/j.cmpb.2019.104986 31443868

[B234] Theilman B. Perks K. Gentner T. Q. (2021). Spike train coactivity encodes learned natural stimulus invariances in songbird auditory cortex. J. Neurosci. 41, 73–88. 10.1523/JNEUROSCI.0248-20.2020 33177068 PMC7786213

[B235] Toosi R. Akhaee M. A. Dehaqani M.-R. (2020). An adaptive detection for automatic spike sorting based on mixture of Skew-t distributions. Sci. Rep. 11, 1–18. 10.1101/2020.06.12.147736 34230517 PMC8260722

[B236] Tóth R. Barth A. M. Domonkos A. Varga V. Somogyvári Z. (2021). Do not waste your electrodes - principles of optimal electrode geometry for spike sorting. J. Neural Eng. 18. 10.1088/1741-2552/ac0f49 34181590

[B237] Tovar K. R. Bridges D. C. Wu B. Randall C. Audouard M. Jang J.. (2018). Action potential propagation recorded from single axonal arbors using multielectrode arrays. J. Neurophysiol. 120, 306–320. 10.1152/jn.00659.2017 29641308

[B238] Tran H. Ranta R. Le Cam S. Louis-Dorr V. (2020). Fast simulation of extracellular action potential signatures based on a morphological filtering approximation. J. Comput. Neurosci. 48, 27–46. 10.1007/s10827-019-00735-3 31953614

[B239] Trautmann E. M. Stavisky S. D. Lahiri S. Ames K. C. Kaufman M. T. Vyas S.. (2019). Accurate estimation of neural population dynamics without spike sorting. Neuron 103, 292–308. 10.1016/j.neuron.2019.05.003 31171448 PMC7002296

[B240] Tsai D. Sawyer D. Bradd A. Yuste R. Shepard K. L. (2017). A very large-scale microelectrode array for cellular-resolution electrophysiology. Nat. Commun. 8. 10.1038/s41467-017-02009-x 29176752 PMC5702607

[B241] Urai A. E. Doiron B. Leifer A. M. Churchland A. K. (2021). Large-scale neural recordings call for new insights to link brain and behavior. Arxiv 1–24. 10.1038/s41593-021-00980-9 34980926

[B242] Valencia D. Alimohammad A. (2019). An efficient hardware architecture for template matching-based spike sorting. IEEE Trans. Biomed. Circuits Syst. 13, 481–492. 10.1109/TBCAS.2019.2907882 30932848

[B243] Valencia D. Alimohammad A. (2021). Neural spike sorting using binarized neural networks. IEEE Trans. Neural Syst. Rehabil. Eng. 29, 206–214. 10.1109/TNSRE.2020.3043403 33296305

[B244] Valencia D. Thies J. Alimohammad A. (2019). Frameworks for efficient brain-computer interfacing. IEEE Trans. Biomed. Circuits Syst. 13, 1714–1722. 10.1109/TBCAS.2019.2947130 31613780

[B245] Vasileva L. N. Bondar I. V. (2021). Long-term stable recording of single-neuron spike activity in the amygdala in conscious rabbits. Neurosci. Behav. Physiol. 51, 322–331. 10.1007/s11055-021-01075-5

[B246] Veerabhadrappa R. Ul Hassan M. Zhang J. Bhatti A. (2020). Compatibility evaluation of clustering algorithms for contemporary extracellular neural spike sorting. Front. Syst. Neurosci. 14, 34. 10.3389/fnsys.2020.00034 32714155 PMC7340107

[B247] Vitale F. Rajagopalan A. Gentile C. (2019). Flattening a hierarchical clustering through active learning. Adv. Neural Inf. Process. Syst. 32. 10.48550/arXiv.1906.09458

[B248] Voitiuk K. Geng J. Keefe M. G. Parks D. F. Sanso S. E. Hawthorne N.. (2021). Light-weight electrophysiology hardware and software platform for cloud-based neural recording experiments. bioRxiv. 18. 10.1088/1741-2552/ac310a 34666315 PMC8667733

[B249] Vu M. A. T. Adali T. Ba D. Buzsáki G. Carlson D. Heller K.. (2018). A shared vision for machine learning in neuroscience. J. Neurosci. 38, 1601–1607. 10.1523/JNEUROSCI.0508-17.2018 29374138 PMC5815449

[B250] Wang M. H. Nikaido K. Kim Y. Ji B. W. Tian H. C. Kang X. Y.. (2017). Flexible cylindrical neural probe with graphene enhanced conductive polymer for multi-mode BCI applications, in 2017 IEEE 30th International Conference on Micro Electro Mechanical Systems (MEMS), 502–505. 10.1109/MEMSYS.2017.7863453

[B251] Wang Q. Yin J. Cui H. (2021). Reinforcement of neuropixels probes for high-density neural recording in non-human primates, in 2021 10th International IEEE/EMBS Conference on Neural Engineering (NER), 128–131. 10.1109/NER49283.2021.9441229

[B252] Weiss M. L. (2019). The autoencoder-kalman filter: theory and practice, in 2019 53rd Asilomar Conference on Signals, Systems, and Computers (Pacific Grove, CA: IEEE), 2176–2179. 10.1109/IEEECONF44664.2019.9048687

[B253] Werner T. Vianello E. Bichler O. Garbin D. Cattaert D. Yvert B.. (2016). Spiking neural networks based on OxRAM synapses for real-time unsupervised spike sorting. Front. Neurosci. 10, 474. 10.3389/fnins.2016.00474 27857680 PMC5093145

[B254] Wood F. Black M. J. Vargas-Irwin C. Fellows M. Donoghue J. P. (2004). On the variability of manual spike sorting. IEEE Trans. Biomed. Eng. 51, 912–918. 10.1109/TBME.2004.826677 15188858

[B255] Wouters F. Kloosterman A. B. (2021). A data-driven spike sorting feature map for resolving spike overlap in the feature space. J. Neural Eng. 18, 12. 10.1088/1741-2552/ac0f4a 34181592

[B256] Wouters J. (2020). Design and validation of low-complexity methods for resolving spike overlap in neuronal spike sorting (Dissertion). KU Leuven, Leuven, Belgium.

[B257] Wouters J. Kloosterman F. (2019). Signal-to-peak-interference ratio maximization with automatic interference weighting for threshold-based spike sorting of high-density neural probe data, in 2019 9th International IEEE/EMBS Conference on Neural Engineering (Leuven: IEEE), 247–250. 10.1109/NER.2019.8716953

[B258] Wouters J. Kloosterman F. Bertrand A. (2020). A Neural Network-based spike sorting feature map that resolves spike overlap in the feature space, in ICASSP 2020 - 2020 IEEE International Conference on Acoustics, Speech and Signal Processing (Barcelona: IEEE), 1175–1179. 10.1109/ICASSP40776.2020.9053530

[B259] Wouters J. Kloosterman F. Bertrand A. (2021). SHYBRID: a graphical tool for generating hybrid ground-truth spiking data for evaluating spike sorting performance. Neuroinformatics 19, 141–158. 10.1007/s12021-020-09474-8 32617751

[B260] Wu H. Yang K. Zeng Y. (2018a). Sparse coding and compressive sensing for overlapping neural spike sorting. IEEE Trans. Neural Syst. Rehabil. Eng. 26, 1516–1525. 10.1109/TNSRE.2018.2848463 29994120

[B261] Wu T. Ratkai A. Schlett K. Grand L. Yang Z. (2019). Learning to sort: few-shot spike sorting with adversarial representation learning, in 2019 41st Annual International Conference of the IEEE Engineering in Medicine and Biology Society (Berlin: IEEE), 713–716. 10.1109/EMBC.2019.8856938 31945996

[B262] Wu T. Zhao W. Keefer E. Yang Z. (2018b). Deep compressive autoencoder for action potential compression in large-scale neural recording. J. Neural Eng. 15. 10.1088/1741-2552/aae18d 30215605

[B263] Xiao Z. Hu S. Zhang Q. (2019). Ensembles of change-point detectors: implications for real-time BMI applications. J. Comput. Neurosci. 46, 107–124. 10.1007/s10827-018-0694-8 30206733 PMC6414295

[B264] Xiong T. Zhang J. Martinez-Rubio C. Thakur C. S. Eskandar E. N. Chin S. P.. (2018). An unsupervised compressed sensing algorithm for multi-channel neural recording and spike sorting. IEEE Trans. Neural Syst. Rehabil. Eng. 26, 1121–1130. 10.1109/TNSRE.2018.2830354 29877836

[B265] Xu F. Zheng Y. Hu X. (2020). Real-time finger force prediction via parallel convolutional neural networks: a preliminary study, in 2020 42nd Annual International Conference of the IEEE Engineering in Medicine & Biology Society (Montreal, QC: IEEE), 3126–3129. 10.1109/EMBC44109.2020.9175390 33018667

[B266] Xu H. Han Y. Han X. Xu J. Lin S. Cheung R. C. C. (2019). Unsupervised and real-time spike sorting chip for neural signal processing in hippocampal prosthesis. J. Neurosci. Methods 311, 111–121. 10.1016/j.jneumeth.2018.10.019 30339881

[B267] Yang Y. Boling S. Mason A. J. (2017). A hardware-efficient scalable spike sorting neural signal processor module for implantable high-channel-count brain machine interfaces. IEEE Trans. Biomed. Circuits Syst. 11, 743–754. 10.1109/TBCAS.2017.2679032 28541908

[B268] Yang Y. Mason A. J. (2017). Frequency band separability feature extraction method with weighted haar wavelet implementation for implantable spike sorting. IEEE Trans. Neural Syst. Rehabil. Eng. 25, 530–538. 10.1109/TNSRE.2016.2590560 27416601

[B269] Yeganegi H. Salami P. Daliri M. R. (2020). A template-based sequential algorithm for online clustering of spikes in extracellular recordings. Cognit. Comput. 12, 542–552. 10.1007/s12559-020-09711-x

[B270] Yger P. Spampinato G. L. B. Esposito E. Lefebvre B. Deny S. Gardella C.. (2018). A spike sorting toolbox for up to thousands of electrodes validated with ground truth recordings *in vitro* and *in vivo*. Elife 7, 1–23. 10.7554/eLife.34518 29557782 PMC5897014

[B271] Yousefi A. Amidi Y. Nazari B. Eden U. T. (2020). Assessing goodness-of-fit in marked point process models of neural population coding via time and rate rescaling. Neural Comput. 32, 2145–2186. 10.1162/neco_a_01321 32946712

[B272] Yu B. Mak T. Smith L. Sun Y. Yakovlev A. Poon C. S. (2011). Memory efficient on-line streaming for multichannel spike train analysis, in 2011 Annual International Conference of the IEEE Engineering in Medicine and Biology Society (Boston, MA: IEEE), 2315–2318. 10.1109/IEMBS.2011.6090648 22254804

[B273] Zamani M. Abdullah S. Demosthenous A. (2020a). Dictionary construction for accurate and low-cost subspace learning in unsupervised spike sorting. Int. J. Simul. Syst. Sci. Technol. 14, 1–6. 10.5013/IJSSST.a.21.02.12

[B274] Zamani M. Demosthenous A. (2015). Power optimization of neural frontend interfaces, in 2015 IEEE International Symposium on Circuits and Systems (Lisbon: IEEE), 3008–3011. 10.1109/ISCAS.2015.7169320

[B275] Zamani M. Jiang D. Demosthenous A. (2018). An adaptive neural spike processor with embedded active learning for improved unsupervised sorting accuracy. IEEE Trans. Biomed. Circuits Syst. 12, 665–676. 10.1109/TBCAS.2018.2825421 29877829

[B276] Zamani M. Sokolic J. Jiang D. Renna F. Rodrigues M. R. D. Demosthenous A. (2020b). Accurate, very low computational complexity spike sorting using unsupervised matched subspace learning, in IEEE Transactions on Biomedical Circuits and Systems (IEEE), 221–231. 10.1109/TBCAS.2020.2969910 32031948

[B277] Zeinolabedin S. M. A. Do A. T. Jeon D. Sylvester D. Kim T. T. H. (2016). A 128-channel spike sorting processor featuring 0.175 μw and 0.0033 mm2 per channel in 65-nm CMOS, in 2016 IEEE Symposium on VLSI Circuits (VLSI-Circuits) (Honolulu, HI: IEEE), 4–5. 10.1109/VLSIC.2016.7573467

[B278] Zhang B. Dai J. Zhang T. (2017). NeoAnalysis: a Python-based toolbox for quick electrophysiological data processing and analysis. Biomed. Eng. Online 16, 1–17. 10.1186/s12938-017-0419-7 29132360 PMC5683334

[B279] Zhang J. Nguyen T. Cogill S. Bhatti A. Luo L. Yang S.. (2018). A review on cluster estimation methods and their application to neural spike data. J. Neural Eng. 15. 10.1088/1741-2552/aab385 29498353

[B280] Zhang Z. Constandinou T. G. (2021a). A robust and automated algorithm that uses single-channel spike sorting to label multi-channel neuropixels data, in 2021 10th International IEEE/EMBS Conference on Neural Engineering (Genoa: IEEE), 783–787. 10.1109/NER49283.2021.9441234

[B281] Zhang Z. Constandinou T. G. (2021b). Adaptive spike detection and hardware optimization towards autonomous, high-channel-count BMIs. J. Neurosci. Methods 354, 109103. 10.1016/j.jneumeth.2021.109103 33617917

[B282] Zhu H. Li X. Sun L. He F. Zhao Z. Luan L.. (2020). Clustering with fast, automated and reproducible assessment applied to longitudinal neural tracking. Arxiv. 10.48550/arXiv.2003.08533

